# Aptamer‐SH2 superbinder‐based targeted therapy for pancreatic ductal adenocarcinoma

**DOI:** 10.1002/ctm2.337

**Published:** 2021-02-26

**Authors:** An‐Dong Liu, Jie Zhou, Xiao‐Yang Bi, Guo‐Qing Hou, Shawn Shun‐Cheng Li, Qing Chen, Hui Xu, Xuan Cao

**Affiliations:** ^1^ Department of Medical Genetics, School of Basic Medicine, Tongji Medical College Huazhong University of Science and Technology Wuhan P. R. China; ^2^ Department of Biochemistry and Molecular Biology, School of Basic Medicine, Tongji Medical College Huazhong University of Science and Technology Wuhan P. R. China; ^3^ Department of Biochemistry, Schulich School of Medicine and Dentistry Western University London Ontario Canada; ^4^ Department of Surgery, Union Hospital, Tongji Medical College Huazhong University of Science and Technology Wuhan P. R. China; ^5^ Ultrastructural Pathology Laboratory, Department of Pathology, School of Basic Medicine, Tongji Medical College Huazhong University of Science and Technology Wuhan P. R. China

**Keywords:** aptamer, cell penetrating peptide, pancreatic ductal adenocarcinoma, phosphotyrosine, SH2 superbinder, targeted therapy

## Abstract

**Background:**

Pancreatic ductal adenocarcinoma (PDAC) exhibits the poorest prognosis of all solid tumors with a 5‐year survival rate of less than 10% and a median survival of 6 months after diagnosis. Numerous targeted agents have been developed and evaluated to improve the survival benefit in patients with PDAC. Unfortunately, most agents have been proven futile mainly owing to the dense stroma and the sophisticated signaling pathways of PDAC. Here, we show the potent effectiveness of Aptamer‐SH2 superbinder‐(Arg)9 conjugate on the treatment of PDAC. In this conjugate, DNA aptamer selected against PDAC cell line confers the function of specifically recognizing and binding to the PDAC cells and activated pancreatic stellate cells (PSCs) in stroma; cell penetrating peptide (Arg)9 facilitates the intracellular delivery of fused proteins; SH2 superbinder conducts the drastic blockade of multiple phosphotyrosines (pY)‐based signaling pathways in tumor cells.

**Methods:**

PDAC‐associated pY were reanalyzed by bioinformatics screen. XQ‐2d and SH2 superbinder‐(Arg)9 were crosslinked with BMH to form XQ‐2d‐SH2 CM‐(Arg)9 conjugate. Immunofluorescence was utilized to assess the potency of the conjugate entering cells. MTT and wound healing assays were performed to evaluate the proliferation or migration of PANC‐1 and BxPC‐3 cells, respectively. Western blot and Pulldown assays revealed that conjugate influenced several pY‐based signaling pathways. Tumor‐bearing mice were used to validate XQ‐2d‐SH2 CM‐(Arg)9, which restrained the growth and metastasis of cancer cells.

**Results:**

XQ‐2d‐His‐SH2 CM‐(Arg)9 conjugate restrained proliferation, invasion, and metastasis of PDAC cells with potent efficacy via blocking the activity of several pY‐related signaling cascades. XQ‐2d‐His‐SH2 CM‐(Arg)9 could eliminate the dense stroma of PDAC and then arrive at tumor tissues.

**Conclusions:**

XQ‐2d‐SH2 CM‐(Arg)9 conjugate may efficiently destroy the pancreatic stroma and show potent antitumor efficacy with minimal toxic effect by regulating tumor cell proliferation and metastasis in vitro and in vivo, which makes it to be a promising targeted therapy of PDAC.

## Graphical abstract

**Schematic diagram of XQ‐2d‐His‐SH2 CM‐(Arg)9 in PDAC cells and PSCs**. XQ‐2d‐His‐SH2 CM‐(Arg)9 could bind and penetrate into PSCs, inactivate PSCs, and decrease ECM secretion. XQ‐2d‐His‐SH2 CM‐(Arg)9 could reach tumor tissues, recognize, and enter into the PDAC cells. XQ‐2d‐His‐SH2 CM‐(Arg)9 could function as a broad‐spectrum inhibitor via capturing pY‐containing proteins and blocking multitude pY‐based signaling pathways in PDAC cells.

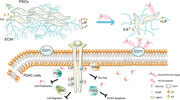

**Abbreviations**
(Arg)9: nine arginines
pY: phosphorylated tyrosine
SH2 CM: SH2 superbinder mutant
SH2 TrM: SH2 superbinder, the Src Homology 2 (SH2) domain with the triple‐mutant
Wt: wild type

## 1 BACKGROUND

Pancreatic adenocarcinoma (PAC) is one of the most lethal cancers with a 5‐year survival rate of less than 10% and a median survival of 6 months after diagnosis.^1^, ^2^ Pancreatic ductal adenocarcinoma (PDAC) accounts for around 95% cases of PAC.^3^ To date, chemotherapy and targeted therapy have failed to improve patient survival significantly in pancreatic cancer.^4^ As the chemotherapeutic agents and the small molecular inhibitors cannot discriminate between the cancerous and the normal cells, most agents may enter into normal cells via the systemic circulation, which causes toxic effect toward normal cells and leads to very low drug concentration around tumor cells, and the case is especially severe in pancreatic cancer owing to the extensive hypovascular stromal reaction.^5^, ^6^

Tyrosine phosphorylation has been proved to be a fundamental mechanism of signal transduction and controls many cellular processes in eukaryotic cells.^7^, ^8^, ^9^ Aberrant tyrosine phosphorylation plays a causal role in cancer.^9^, ^10^ A number of tumor‐targeted drugs, small molecular inhibitors, or monoclonal antibodies, have been evaluated and approved for clinical use in several tumors.^9^, ^11^ Most of them are developed against receptor tyrosine kinases (RTKs). However, erlotinib, one small molecular inhibitor for epidermal growth factor receptor (EGFR), is the only targeted therapy for the clinical treatment of metastasis PDAC, just providing a marginal survival benefit of approximately 2 weeks.^12^

There is emerging evidence for redundancy among RTKs involved in cancer.^9^, ^13^, ^14^ Inhibition of one RTK may result in compensatory activation of other ones.^15^ Signaling systems often involve a cascade mechanism of sequential phosphorylation of signaling proteins. Aberrant high levels of phosphotyrosine (pY) proteins were reported to play a critical role in PDAC carcinogenesis.^16^, ^17^, ^18^ Meanwhile, many cytoplasmic tyrosine kinases (CTKs) take part in relaying signals through phosphorylating proteins in the cascades.^19^, ^20^, ^21^ Hence, it is not hard to explicate why pharmacological inhibition of RTKs only represent negligible effect. The combination use of several single‐target tyrosine kinase inhibitors (TKIs) or multikinase inhibitors may, to some extent, meliorate the efficacy.^9^ Undoubtedly, this means is impossible to block the action of numerous RTKs and CTKs in the complicated signaling cascades, so its efficacy must be limited. Novel targeted therapy strategy is urgently needed for pancreatic cancer.

The Src homology 2 (SH2) domain can specifically recognize and bind to the pY residues. SH2‐containing proteins play a vital role in the pY‐based signal transduction.^7^ Generally, the natural SH2 domains present moderate affinity to pY residues and have different pY sequence binding preferences. The SH2 domain with triple mutants, called SH2 superbinder herein, was identified to have stronger affinity to pY residues than the natural counterpart and might efficiently capture diverse pY peptides.^22^, ^23^, ^24^, ^25^ Especially, the minor difference of pY sequence context was verified to have no obvious effect on the binding preference under condition of excess SH2 superbinders.^25^ After introduced into tumor cells, SH2 superbinder would displace the endogenous SH2 domain‐containing proteins and block a large number of pY signaling pathways.^26^, ^27^

In the present work, we utilize SH2 superbinder as the broad‐spectrum inhibitor for multitude pY‐based signaling pathways. To facilitate the membrane penetration, (Arg)9, one cell penetrating peptide (CPP),^28^ has been fused with SH2 superbinder protein. XQ‐2d aptamer selected against PDAC cell line has been identified to be able to specifically recognize and bind the target molecule, transferrinreceptor1 (TfR1, CD71), on the cell surface of PDAC cells.^29^ We conjugate PDAC‐specific aptamer with the fusion protein (Arg)9‐SH2 superbinder, thereby, making this complex accurately aim at, efficiently enter into, and dramatically destroy the activated pancreatic stellate cells (PSCs) and PDAC cells by blocking their pY‐based signaling pathways.

## 2 MATERIALS AND METHODS

### 2.1 Plasmids and protein purification

(Arg)9 was subcloned into pETM11‐SH2 TrM plasmid. The pETM11‐Src SH2 was digested with NcoI (cat. # R0193V) and XhoI (cat. # R0146V). With the help of One Step Cloning Kit, purified PCR products were linked with linearized pETM11‐SH2 TrM. Then, pETM11‐SH2 CM‐(Arg)9 was constructed by Fast Site‐Directed Mutagenesis Kit (KM101, TIANGEN) and verified by DNA sequencing. Sequences of amino acids and primers are shown in Supporting information Tables S1 and S2.

His‐tagged fusion proteins were expressed, purified, and identified according to previous procedures.^27^ BCA method was used to determine the concentrations of the purified proteins.

### 2.2 Chemical cross‐linking

5′‐SH‐XQ‐2d(5′‐3′:‐SH‐ACTCATAGGGTTAGGGGCTGCTGGCCAGATACTCAGATGGTAGGGTTACTAT, Sangon Biotech) and BMH (dissolved in DMSO at 10 mM concentration) were reacted. Then the His‐SH2 CM‐(Arg)9 (diluted in PBS) was added into the compounds at 4°C for 2 h, and then SDS sample buffer was added. Detailed procedure for crosslinking can refer to the instruction of BMH (Thermo Scientific, 22330). Samples were analyzed by silver staining and western blot with the antibody against His‐tag.

### 2.3 Cell culture and administration

BxPC‐3, PANC‐1, SW1990, MDA‐MB‐231, MCF7, hTERT‐HPNE, and MiaPaca‐2 were obtained from ATCC. PL45 and H1299 cell lines were generous gifts from Professor Mao Ye (Hunan University). The human pancreatic duct epithelial (HPDE) cells were obtained from patients with PDAC who underwent surgical resection in the Wuhan Union Hospital. PL45, H1299, BxPC‐3, PANC‐1, SW1990, MDA‐MB‐231, and MCF7 cells were cultured in DMEM medium supplemented with 10% fetal bovine serum and 100 U/mL penicillin–streptomycin. The culture conditions of hTERT‐HPNE cells and the primary HPDE cells followed the procedures reported formerly.^30^, ^31^ PSCs of human were from pancreatic cancer surgical patients with the method previously reported.^32^, ^33^ All experiments with PSCs were performed between passages 3 and 8. All cell lines were cultured with 5% CO_2_ at 37°C.

For protein extraction, cells were kept hunger for 6 h before collection. The whole cell lysates (WCL) of PANC‐1 cells was incubated with His‐tagged fusion proteins or aptamer, and the appropriate amount of Ni‐NTA beads was added. After centrifugation, discard the supernatant and collect the proteins.

### 2.4 Western blot and pulldown assay

Cells were lysed on ice using lysis buffer as instructed before^27^; then the lysate proteins were collected and further analyzed. Protein A/G agarose or Ni‐NTA beads were used for immunoprecipitation or pulldown assays, respectively. Immunoblotting was performed according to standard method with primary antibodies against pY antibody (Abcam EPR16871), pEGFR (CST#3777S), EGFR (CST#4267), pSTAT3 (CST#4113), STAT3 (CST#9139), CK8 (17514‐1‐AP, Proteintech), CD71 (ABclonal#A5865), Vimentin (10366‐1‐AP, Proteintech), GRB2 (CST#3972), Snail1 (ABclonal#A5243), pIGF‐1R (CST#3021), IGF‐1R (20254‐1‐AP, Proteintech), IRS‐1 (17509‐1‐AP, Proteintech), pVEGFR2 (CST#3817S), VEGFR2 (CST#9698), SHC (10054‐1‐AP, Proteintech), pAKT (CST#4060), pERK1/2 (CST#4370), pJAK1 (CST#3331), AKT (CST#9272), N‐cadherin (22018‐1‐AP, Proteintech), ERK1/2 (CST#4695), JAK1 (CST#3344), LIFR (22779‐1‐AP, Proteintech), His (CST#12698), GP130 (21175‐1‐AP, Proteintech), αSMA (55135‐1‐AP, Proteintech), E‐cadherin (20874‐1‐AP, Proteintech), Smad2/3 (CST#8685), Fibronectin (15613‐1‐AP, Proteintech), FAP (ABclonal#A6349), collagen‐I (14695‐1‐AP, Proteintech), pSmad2/3 (CST#8828), TWIST1 (25465‐1‐AP, Proteintech), and GAPDH (CST#5174) were used at recommended dilutions followed by HRP‐conjugated antibodies and analyzed with the ECL system.

### 2.5 Immunofluorescent staining

Cells were incubated with XQ‐2d‐His‐SH2 CM‐(Arg)9 with indicated conditions. Cells were then fixed with 4% formaldehyde and then permeabilized with 1% tritonX‐100. Next step, anti‐His‐tag antibody and goat‐anti‐rabbit FITC secondary antibody were used to stain cells. After incubated with rhodamine phalloidin (Invitrogen, 1:50) for 30 min, samples were stained with DAPI for 5 min and visualized by an immunofluorescence microscopy (Olympus, Japan).

### 2.6 qRT‐PCR

The assays were performed as previously described.^34^ In brief, total RNA from cells was extracted and reverse transcription assay was carried out using Reverse Transcription Kit (Toyobo). *Ct* (2^–ΔΔ^ *^Ct^*) value for each sample was normalized to the value for GAPDH gene. Sequences of all primer for CD71, TGF‐β, TNF‐α, and VEGF‐A genes are shown in Supporting information Table S3.

### 2.7 MTT and colony formation assay

For MTT assays, cells in 96‐well plate were treated with XQ‐2d‐His‐SH2 CM‐(Arg)9 with different concentrations or incubation time according to the experiment design. The optical absorbance was measured at the wavelength of 570 nm using an ELISA reader (Thermo Fisher).

For colony formation assays, cells in 12‐well plates (100 cells/well) were stimulated with XQ‐2d‐His‐SH2 CM‐(Arg)9 and other negative controls. The numbers of colonies were counted at three different wells under a microscope (Mshot, Guangzhou, China). Details were shown as previous study.^27^

### 2.8 Wound healing assay and transwell assay

For wound healing assays, detailed procedures could follow previous study.^35^ Images were taken with a digital camera at different time points under Inverted Microscope (Mshot).

For transwell assays, XQ‐2‐His‐SH2 CM‐(Arg)9 was added to the upper chamber and cells were allowed to invade the Matrigel (BD Biosciences) coated membranes. Following procedures could be done as previously reported.^27^ Numbers of invasion cells were counted at three different areas under a microscope.

### 2.9 siRNA treatment

The oligonucleotides targeting CD71 mRNA (5′‐GAACUUGAAACUGCGUAAATT‐3′) and negative control (5′‐UCUUAAUCGCGUAUAAGGCTT‐3′)^36^ were synthesized and transfected into cells with Lipofectamine RNAiMAX reagent (Invitrogen) according to the manufacturer's protocol.

### 2.10 Animal studies

All animal experiments were approved by the Institutional Animal Care and Use Committee of Tongji Medical College, Huazhong University of Science and Technology. PANC‐1 cells (1 × 10^6^ cells for each mouse) were inoculated into nude mice. When the volume of tumors reached about 200 to 300 mm^3^, all mice would be separated into nine groups randomly (n = 10). His, (Arg)9, His‐SH2 CM, His‐(Arg)9, XQ‐2d, His‐SH2 CM, His‐SH2 CM‐(Arg)9, XQ‐2d‐His‐SH2 CM, XQ‐2d‐His‐SH2 CM‐(Arg)9, and PBS were injected into the tail vein every day, respectively. *V* = 1/2 × *W* ^2^ × *L* (*V* is volume, *L* is length, and *W* is width). All animals would be sacrificed when the tumor size reached about 1000 mm^3^.

For the PANC‐1 metastasis mouse model, 5 × 10^5^ cells (in 100 μL PBS) were inoculated s.c. into nude mice through tail veil, as previously reported.^37^ Mice were randomly separated into two groups (n = 5). XQ‐2d‐His‐SH2 CM‐(Arg)9 and PBS were injected through tail vein once every 2 days, respectively.

### 2.11 Hematology analysis and blood biochemical assay

ALT, LDH, AST, and TBIL were assayed in serum, following the instructions (Nanjing Jiancheng Corp.). Blood routine tests were performed at the Servicebio Company, Wuhan.

### 2.12 Bone marrow isolation

Bone marrow of mice came from their hind limb long bones and details can refer to the previous protocol.^38^

### 2.13 Transmission electron microscopy

Small intestines from different groups were fixed with 2.5% glutaraldehyde solution according to the previous description.^39^ Images were captured by a transmission electron microscope (JEOL, Japan).

### 2.14 ELISA assay

Leukemia Inhibitory factor (LIF) in PSC culture medium was evaluated by using a human LIF ELISA kit (DLF00B). LIF, IL6, and IL11 in mouse tissues or serum were measured by using mouse ELISA kits (MLF00, M6000B, and DY418). All ELISA kits were from R&D Systems and procedures were conducted according to the instructions.

### 2.15 IHC assay, HE staining, and TUNEL assay

These assays were conducted as previously described.^27^ Tumor sections were stained with indicated antibodies for IHC assays. TUNEL assay kit was used in TUNEL assays. Images were taken with a microscope (Mshot).

### 2.16 Data analysis and presentation

MS datasets^40^ of normal pancreatic cell and different pancreatic cancer cell lines were reanalyzed for tyrosine phosphorylation levels using TB tools software. Hierarchical clustering was performed in Persues using Euclidian distance and average linkage clustering.

### 2.17 Patients and sample collection

PDAC specimens and the adjacent parts were obtained from patients who had undergone surgical resection for PDAC at Wuhan Union Hospital and Wuhan Tongji Hospital. Tissue acquisition and handling of human tissue specimens used in this study have been approved by the Ethics Committee of Tongji Medical College, Huazhong University of Science and Technology.

### 2.18 Statistical analysis

Results are presented as mean ± standard deviation (SD) and analyzed, using Student's *t*‐test (two groups) or one‐way ANOVA (more than two groups) if they complied with normal distribution. *P* value less than 0.05 was considered as statistically significant.

## 3 RESULTS

### 3.1 High tyrosine phosphorylation levels in tumors of PDAC patients and several cell lines

**FIGURE 1:**
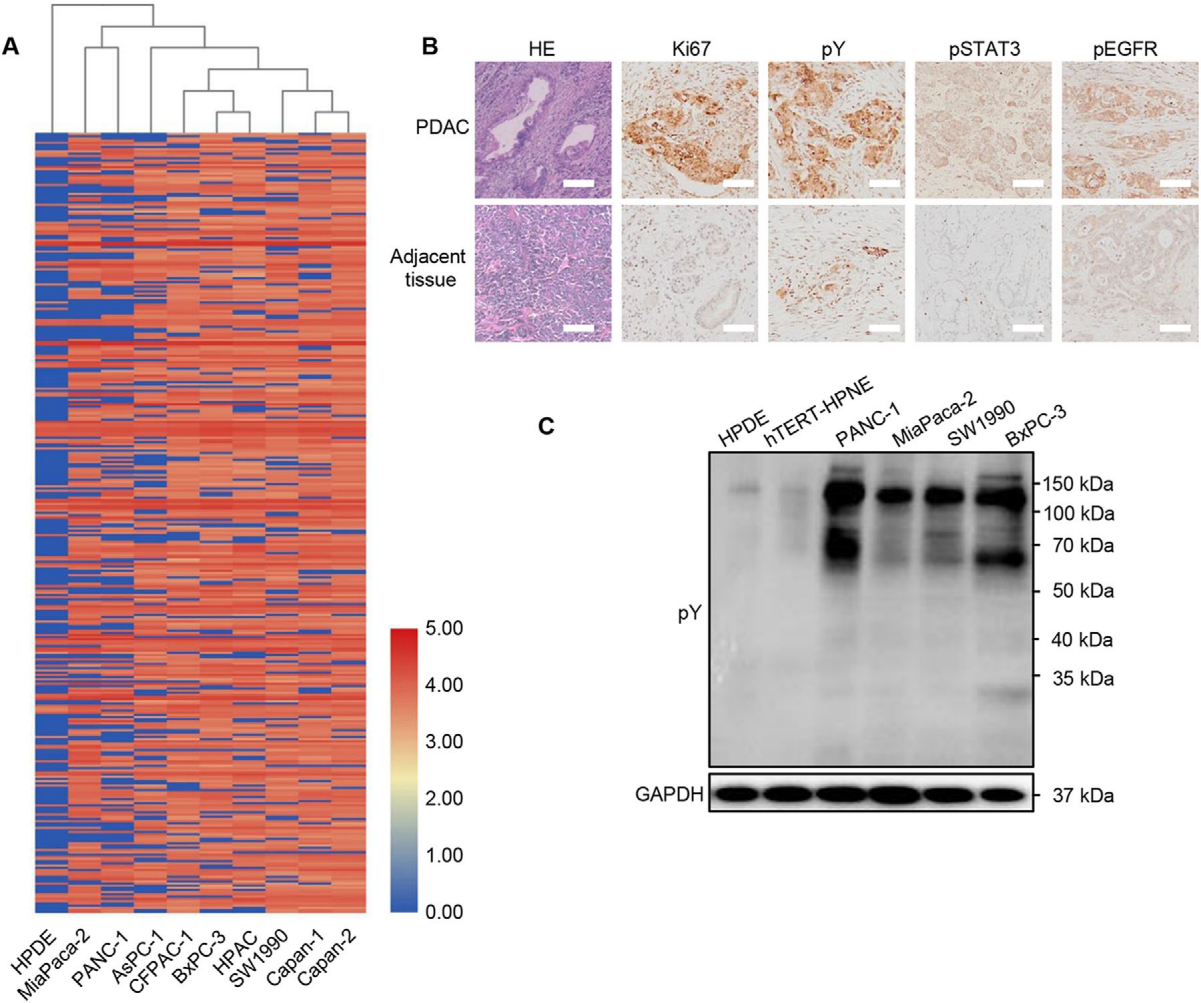
High phosphotyrosine levels in tumors of PDAC patients and cell lines. (A) The hierarchical clustering of specific tyrosine sites with high relative phosphorylation of one normal pancreatic cell line and nine PDAC cell lines. (B) Representative histological examinations of the dissected tumors using HE staining, Ki67, pY, pSTAT3, and pEGFR staining. Little pY, pEGFR, or pSTAT3 was present in normal tissue adjacent to the tumors, whereas there was an enhanced phosphotyrosine level of these proteins within tumor tissues (Scale bar: 50 μm). (C) The global levels of phosphotyrosine‐containing proteins in PDAC cells and normal pancreatic cells. The results are representative of three independent experiments with similar results

In PDAC, constitutive activation of several proteins by phosphorylation of tyrosine has been reported in human specimens and PDAC cell lines such as STAT3, EGFR, and IGF‐1R.^41^, ^42^, ^43^ Aberrant activation of these phosphorylated tyrosine (pY) proteins plays an essential role in PDAC carcinogenesis. Global tyrosine phosphorylation patterns were characterized across two large panels of human PDAC cell lines: the ATCC series (19 cell lines) and TKCC series (17 cell lines) by using immunoaffinity‐coupled high‐resolution mass spectrometry.^40^ To confirm phosphorylation of tyrosine in pancreatic cancer, we reanalyzed data with one normal pancreatic cell (HPDE) and nine pancreatic cancer cell lines. The levels of pYs in multiple proteins were significantly elevated in pancreatic cancer cell lines (Figure 1A, details of pYs are shown in Supporting information Data S1) including EGFR, ERBB3, and MET.^40^ To further investigate the significance of the pYs‐related signaling pathways in human pancreatic cancers, we measured the phosphorylation levels of total Tyrosine, EGFR, and STAT3 in PDAC and adjacent tissues in patients who have received surgical resection of their tumors (Figure 1B). While little pY, pEGFR or pSTAT3 was present in normal tissue adjacent to the tumors, there was an enhanced level of pY, pEGFR, or pSTAT3 within tumor tissues. Consistent with these results, the expression of Ki67 was higher in tumor tissues than the adjacent part based on the diagnosis of the pathologist. Furthermore, we detected the pY levels in PDAC and normal pancreatic cell lines, and the data demonstrated that phosphorylation level of tyrosine in PDAC cells is much higher than that in normal pancreatic cells (Figure 1C).

### 3.2 XQ‐2d‐His‐SH2 CM‐(Arg)9 conjugate could specifically enter into PDAC cells

**FIGURE 2:**
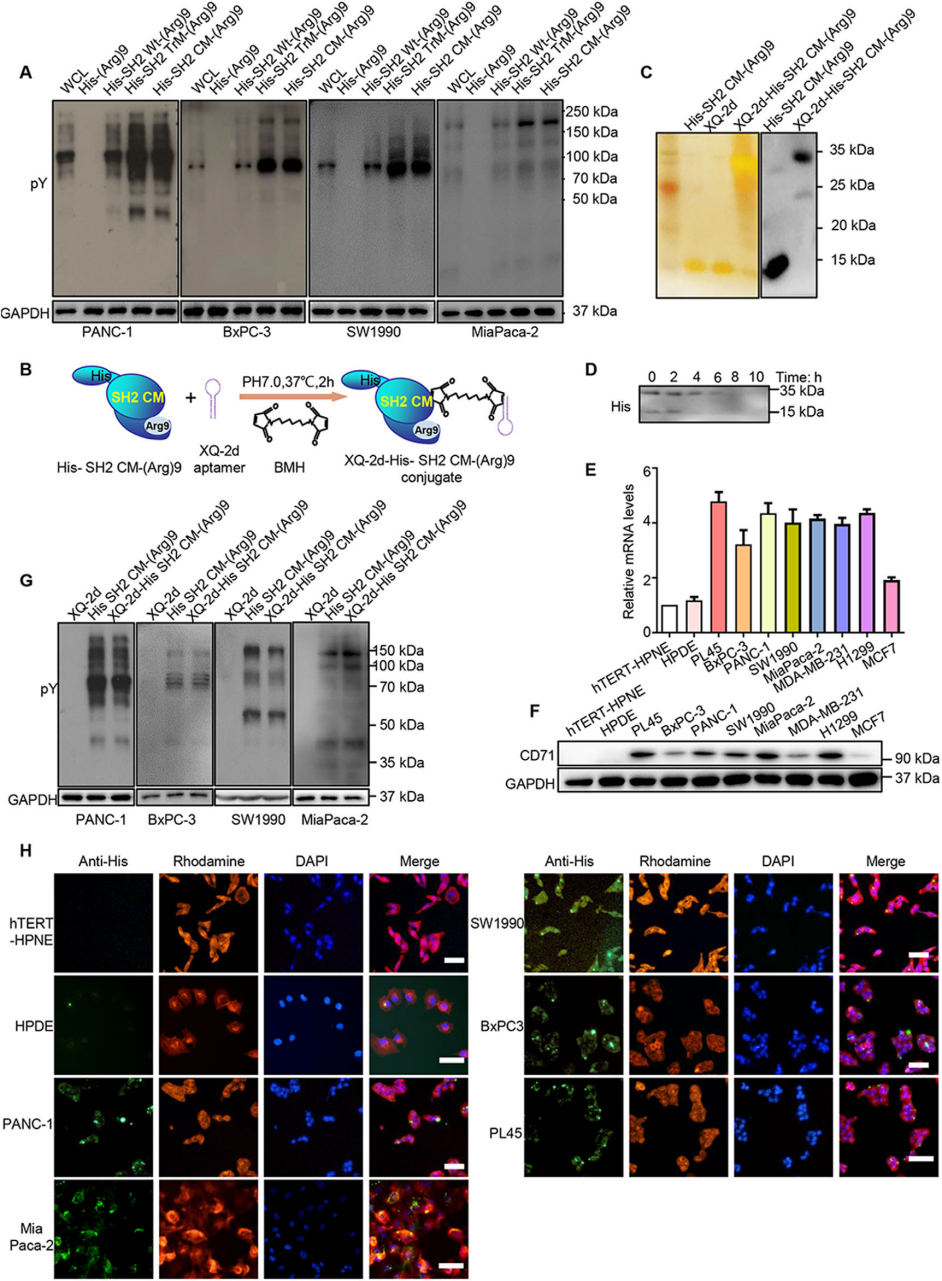
XQ‐2d‐His‐SH2 CM‐(Arg)9 could effectively bind with pY proteins and specifically enter into PDAC cells. (A) The pY proteins were enriched and pulled down from PANC‐1, BxPC‐3, SW1990, and MiaPaca‐2 cells. The whole cell lysates were incubated with His‐(Arg)9, His‐SH2 Wt‐(Arg)9, His‐SH2 TrM‐(Arg)9, or His‐SH2 CM‐(Arg)9 overnight at 4°C. The data demonstrated cellular pY proteins by pervanadate administration with antiphosphotyrosine antibody. (B) Schematic diagram of XQ‐2d‐His‐SH2 CM‐(Arg)9 conjugate. BMH, a crosslinker for covalent, was employed between sulfhydryl groups on Cys130 of His‐SH2 CM‐(Arg)9 and 3′ terminus of XQ‐2d aptamer. (C) Results of the silver staining and western blot showing the XQ‐2d‐His‐SH2 CM‐(Arg)9 conjugate. (D) Degradation of XQ‐2d‐His‐SH2 CM‐(Arg)9 conjugate in cell culture medium. (E, F) Expression of CD71 on mRNA and protein levels of several cancer cell lines and normal cell lines. (G) XQ‐2d‐His‐SH2 CM‐(Arg)9 showed similar strongly binding capacity with pY‐containing proteins as His‐SH2 CM‐(Arg)9 in PANC‐1, BxPC‐3, SW1990, and MiaPaca‐2 cells. (H) Several PDAC cells and normal cell lines were incubated with 100 nM XQ‐2d‐His‐SH2 CM‐(Arg)9 for 3 h, the fluorescence signal was detected by immunofluorescence assay. Scale bar: 20 μm. All images shown are representative of at least three independent experiments

Recently, we have identified SH2 superbinder presented strong affinity to pY residues and showed potent antitumor activity by displacing proteins containing Wild type (Wt) SH2 domain and blocking the pathways based on pY signaling.^22^, ^44^ To help transporting therapeutic drug across cell membranes, oligoarginines, one kind of the CPPs, have been utilized here. The recombinant protein was constructed based on the His‐tagged Src SH2 domain triple mutant protein with (Arg)9 at C‐terminus, named His‐SH2 TrM‐(Arg)9 (Supporting information Tables S1 and S2). Fusion proteins were largely expressed and purified with Ni‐NTA Agarose (Supporting information Figure S1). Src SH2 TrM protein comprises two Cysteine residues at its C‐terminus on site 123 and 130 (Supporting information Table S1). To cross‐link with aptamer specifically in next step, we mutated the Cysteine residue on site 123 to Serine without affecting the structure or ligand‐binding properties^45^ (Figure 2A and B), and the modified SH2 superbinder was named SH2 CM. The WCLs were incubated with His‐(Arg)9, His‐SH2 Wt‐(Arg)9, His‐SH2 TrM‐(Arg)9, or His‐SH2 CM‐(Arg)9. Then, the WCLs were enriched and pulled down by Ni‐NTA agarose. The results displayed that there is almost no band in His‐(Arg)9‐treated group, whereas bands of pY‐containing proteins from His‐SH2 TrM‐(Arg)9‐ or His‐SH2 CM‐(Arg)9‐treated group were similar and more obvious than that from His‐SH2 Wt‐(Arg)9‐treated group in pancreatic cancer cell lines (Figure 2A).

**FIGURE 3:**
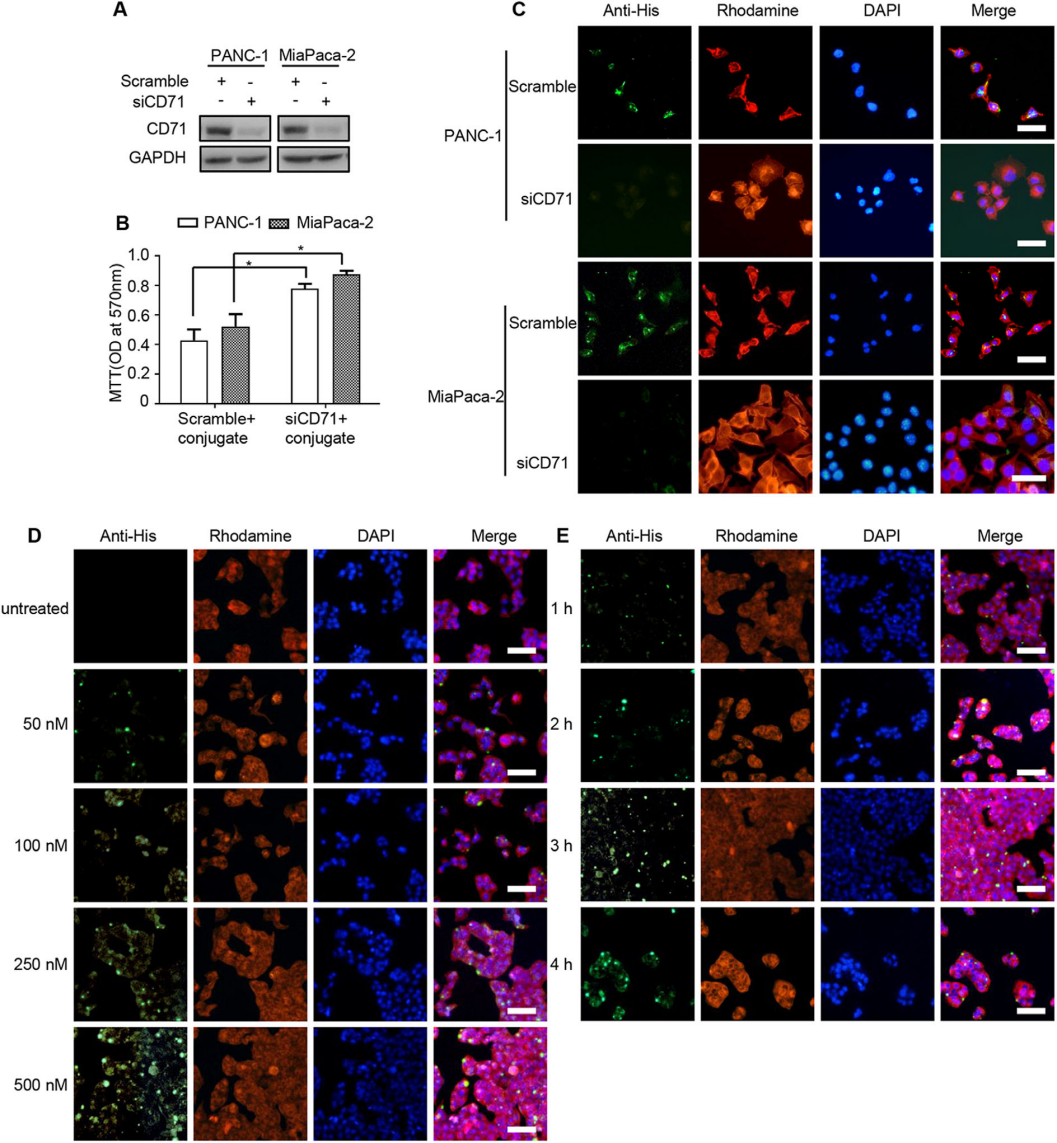
XQ‐2d‐SH2 CM‐(Arg)9 could efficiently penetrate into PANC‐1 and MiaPaca‐2 cells. (A) PANC‐1 and MiaPaca‐2 cells were transfected with indicated siRNAs for 24 h, and samples were collected and analyzed by western blotting with anti‐CD71 antibody. (B) MTT assays were conducted to evaluate the inhibitory ability of conjugate in PANC‐1 and MiaPaca‐2 cells with siRNAs. (C) Immunofluorescence staining assays were performed to assess the penetration ability of conjugate in PANC‐1 and MiaPaca‐2 cells with siRNAs. Effects of XQ‐2d‐SH2 CM‐(Arg)9 translocating into PANC‐1 cells at various concentrations (50, 100, 250, and 500 nM) (D) and time points (1, 2, 3, and 4 h) (E). Cells were stained with anti‐His antibody, followed by goat‐anti‐rabbit FITC secondary antibody. Actin was labelled with Rhodamine‐phallodin (Red) and nucleus with DAPI (Blue). Scale bar: 20 μm. All images shown are representative of at least three independent experiments

Oligoarginines confer the (Arg)9‐fused proteins the excellent cell membrane penetrating ability, however, it lacks the function of distinguishing tumor cells from normal ones. To overcome this shortcoming, the aptamer technique was adopted.^46^, ^47^ XQ‐2d, a DNA aptamer selected against PDAC cell line, has been reported to specifically recognize and bind to PDAC cells by targeting CD71 on the cell surface, not to the normal counterpart.^29^, ^30^ A sulfhydryl group (‐SH) was added at the 3′ terminus of XQ‐2d aptamer. We employed the BMH, a cross‐linker for covalent, irreversible conjugation between sulfhydryl groups on Cysteine130 of SH2 CM and 3′ terminus of XQ‐2d aptamer (Figure 2B). Molecular weights of His‐SH2 CM‐(Arg)9, XQ‐2d, and XQ‐2d‐His‐SH2 CM‐(Arg)9 were about 14, 14, and 32 kDa, respectively (Figure 2C). Then degradation of XQ‐2d‐His‐SH2 CM‐(Arg)9 in the DMEM medium was examined by western blot and the band would disappear at about 6 h (Figure 2D). To confirm the expression of target protein CD71, we examined it on RNA and protein levels of several cell lines. The cancer cell lines contained PDAC cell lines (PL45, BxPC‐3, PANC‐1, MiaPaca‐2, and SW1990) and three cancer cell lines used in the previous research (H1299, MDA‐MB‐231, and MCF7). The expression levels of CD71 were high in PDAC cells, H1299, and MDA‐MB‐231, whereas the levels were low in MCF7 and the normal cell lines (hTERT‐HPNE and HPDE) showed no obvious expression (Figure 2E and F), which were consistent with previous reports.^48^, ^49^ Meanwhile, PANC‐1 and MiaPaca‐2 cells transfected with siRNA targeting CD71 displayed enhanced cell viability (Figure 3A and B) and conjugate failed to enter into them (Figure 3C). Considering that many modifications were made on SH2 superbinder, we investigated whether the XQ‐2d‐His‐SH2 CM‐(Arg)9 conjugate would retain high binding ability to pY‐containing proteins under native conditions. Fortunately, XQ‐2d‐His‐SH2 CM‐(Arg)9 conjugate was found to show similar strong binding capacity with pY‐containing proteins as His‐SH2 CM‐(Arg)9, whereas the XQ‐2d only pulled down little pY protein (Figure 2G).

We anticipate that the conjugate of XQ‐2d aptamer and fusion protein His‐SH2 CM‐(Arg)9 may precisely aim at and then efficiently enter into PDAC cells, not into the normal cells. To validate this assumption, we incubated the conjugate at different concentrations with several cell lines. For PANC‐1 cells, the conjugate from 500 to 100 nM could be observed to distribute in the cells, while there was little fluorescence in the 50 nM group (Figure 3D). And the intracellular fluorescence signal increased with the incubation time prolonged (Figure 3E). Whereas, for the normal cell line hTERT‐HPNE, only very weak signal of the conjugate at 250 nM was observed and no signal was observed at 100 nM and below when the incubation time was 3 h (Supporting information Figure S2A). Meanwhile, the hTERT‐HPNE cell line and several PDAC cells were incubated with 100 nM XQ‐2d‐His‐SH2 CM‐(Arg)9 for 3 h, the fluorescence signal was detected by immunofluorescence assay. The data showed that XQ‐2d‐His‐SH2 CM‐(Arg)9 could only enter into PDAC cells, H1299, and MDA‐MB‐231 cells, not into hTERT‐HPNE, HPDE, or MCF7 cells (Figure 2H, Supporting information Figure S2B). These solid data demonstrated that XQ‐2d aptamer has binary functions: when incubated with PDAC cells, it helps the conjugate translocate into cells by specifically recognizing and binding them; when incubated with the normal cells, it restrains the conjugate from approaching and contacting the cells for the steric hindrance.

### 3.3 XQ‐2d‐His‐SH2 CM‐(Arg)9 conjugate exhibited potent antitumor efficacy in vitro

**FIGURE 4:**
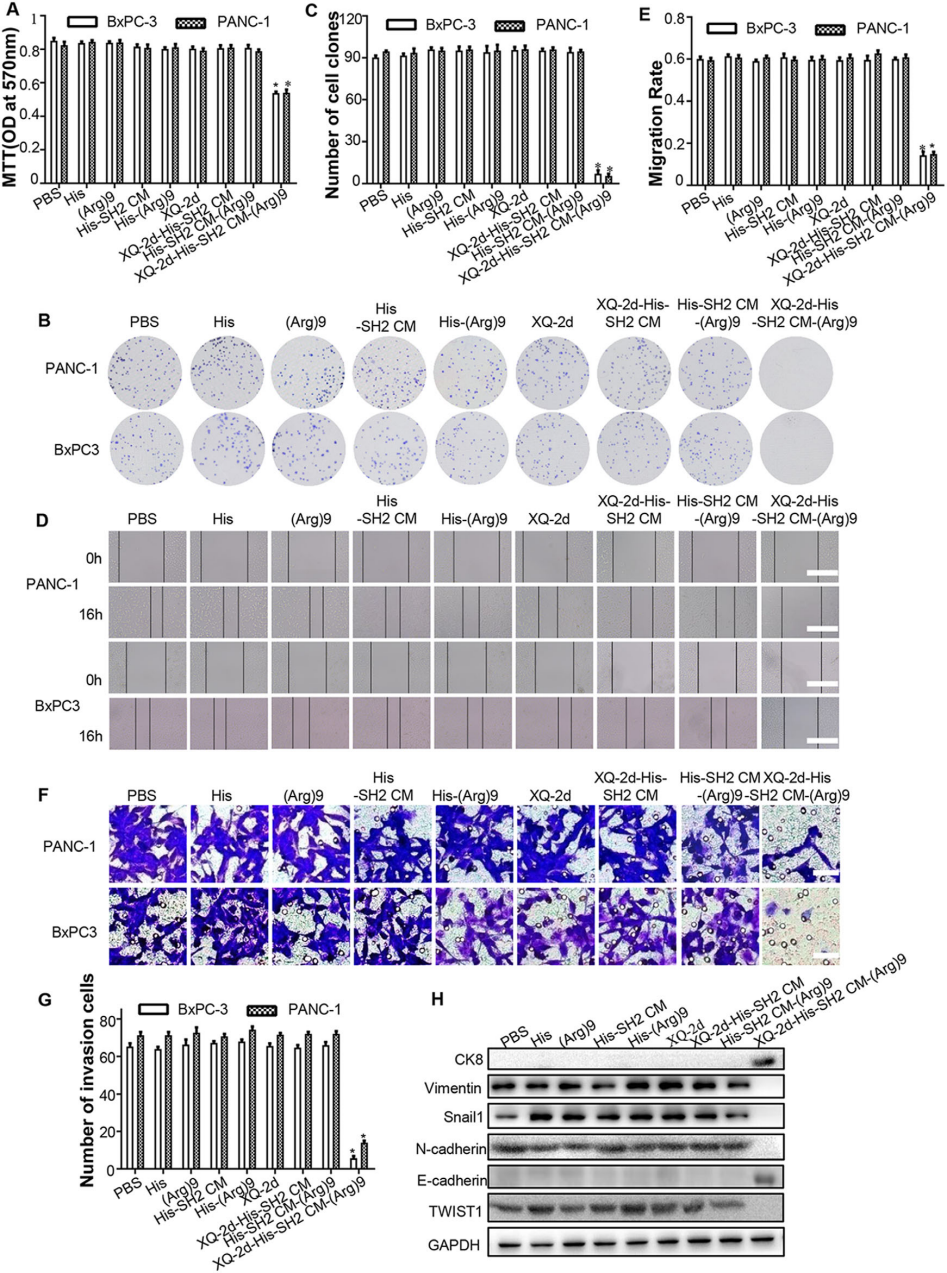
XQ‐2d‐His‐SH2 CM‐(Arg)9 conjugate exhibited potent antitumor efficacy in vitro. (A) Effects of XQ‐2d‐His‐SH2 CM‐(Arg)9 on the proliferation of BxPC‐3 and PANC‐1 cells. Cells were treated with His‐alone, (Arg)9, His‐SH2 CM, His‐(Arg)9, XQ‐2d, XQ‐2d‐His‐SH2 CM, XQ‐2d‐His‐SH2 CM‐(Arg)9, and His‐SH2 CM‐(Arg)9 at 100 nM for 3 h. Cell viability was measured by MTT assays. (B) Representative images of colony formation assay showing colonies formed by cells incubated with different treatments. (C) Bar graph depicting the change in the number of cell colonies. (D) Wound healing assays were monitored at 0 and 16 h in PANC‐1 or BxPC‐3 cells with different treatments. Scale bar: 100 μm. (E) Bar graph depicting the change of migration rate. (F) Representative images and results of transwell assays of PANC‐1 or BxPC‐3 cells with different treatments. Scale bar: 20 μm. (G) Bar graph depicting the change in the number of invasion cells. (H) The expressions of N‐cadherin, E‐cadherin, Vimentin, CK8, TWIST1, and Snail1 were examined by western blot. Cells were stimulated with XQ‐2d‐His‐SH2 CM‐(Arg)9 and other negative controls at 100 nM for 3 h. All images shown are representative of at least three independent experiments (*P < 0.05)

As XQ‐2d‐His‐SH2 CM‐(Arg)9 showed strong affinity to PDAC cells, we investigated the cytotoxicity of the conjugate in vitro. The antitumor efficacy of XQ‐2d‐His‐SH2 CM‐(Arg)9 conjugate against PANC‐1 and BxPC‐3 cells was evaluated by MTT, colony formation, matrigel invasion, and wound healing assays. PANC‐1 cells were treated with different concentrations or incubation time periods to determine the optimal conditions in preliminary experiments and the results are shown in Supporting information Figure S3A and S3B. As illustrated by MTT assays, XQ‐2d‐His‐SH2 CM‐(Arg)9 conjugate demonstrated potent inhibitory effects, while control groups, such as His, (Arg)9, His‐SH2 CM, His‐(Arg)9, XQ‐2d, His‐SH2 CM, His‐SH2 CM‐(Arg)9, and XQ‐2d‐His‐SH2 CM, had no obvious cytotoxicity effects to PANC‐1, BxPC‐3, or MiaPaca‐2 cells (Figure 4A, Supporting information Figure S3C). Consistent with the results of MTT assays, colony formation analysis showed that the cell colonies in XQ‐2d‐His‐SH2 CM‐(Arg)9 conjugate treated groups were almost completely eliminated (Figure 4B and C, Supporting information Figure S3D and E). These results demonstrated a potent long‐term inhibitory effect of the conjugate against PDAC cells. Meanwhile, to verify the cytotoxicity in normal cells, MTT and colony formation assays were performed. The data showed XQ‐2d‐His‐SH2 CM‐(Arg)9 had no obvious cytotoxicity effect on hTERT‐HPNE or HPDE cells (Supporting information Figure S3F‐S3H).

To examine whether XQ‐2d‐His‐SH2 CM‐(Arg)9 would inhibit the migration of PDAC cells, wound healing assay was conducted. Cells were seeded in six‐well plates, and cultured with complete cell medium or contained with 100 nM different fusion proteins. PDAC cells treated with XQ‐2d‐His‐SH2 CM‐(Arg)9 conjugate had limited ability of migration after scratched for 16 h (Figure 4D and E, Supporting information Figure S3I and J). Furthermore, to characterize the effects of XQ‐2d‐His‐SH2 CM‐(Arg)9 conjugate on the invasion of PANC‐1, BxPC‐3, or MiaPaca‐2 cells, we conducted matrigel invasion assays. Compared with the PBS group, significantly fewer PANC‐1, BxPC‐3, or MiaPaca‐2 cells treated with XQ‐2d‐His‐SH2 CM‐(Arg)9 conjugate invaded the lower surface of the chamber (Figure 4F and G, Supporting information Figure S3K and L). Meanwhile, some epithelial‐mesenchymal transition (EMT)‐related markers (Vimentin, CK8, and Snail1) were assessed in PANC‐1 cells. The results proved that CK8 and E‐cadherin increased after the conjugate treatment, whereas Vimentin, *N*‐cadherin, Snail1, and TWIST1 decreased at the same time (Figure 4H), revealing that SH2 superbinder could impair the progression of EMT. Taken together, these data indicated that the migration and invasion abilities of PDAC cells were attenuated by XQ‐2d‐His‐SH2 CM‐(Arg)9.

### 3.4 XQ‐2d‐His‐SH2 CM‐(Arg)9 influenced multiple signaling cascades of PDAC cells

**FIGURE 5:**
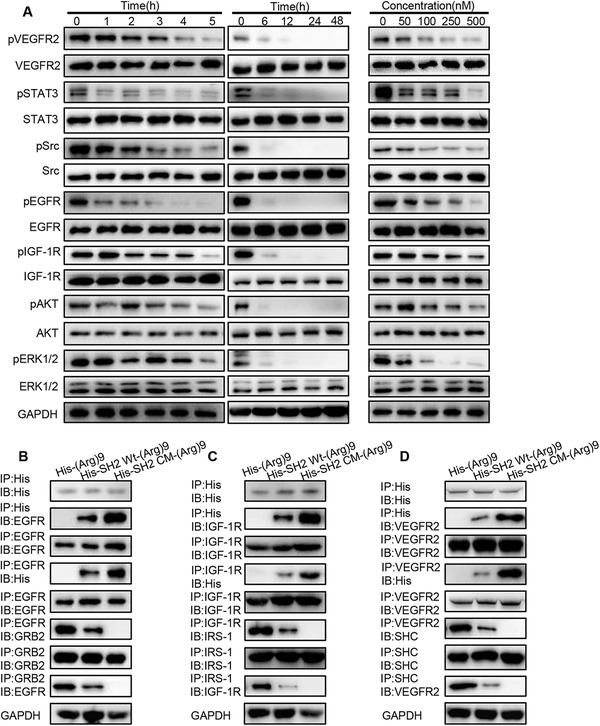
XQ‐2d‐His‐SH2 CM‐(Arg)9 influenced multiple signaling cascades of PANC‐1 cells. (A) Inhibition of EGFR, VEGFR2, IGF‐1R, Src, AKT, and ERK1/2 phosphorylation by XQ‐2d‐His‐SH2 CM‐(Arg)9 at different incubation time or concentrations was examined in PANC‐1 cells. (B‐D) The panels showed reciprocal immunoprecipitation of EGFR and GRB2, IGF‐1R and IRS1, VEGFR2, and SHC in PANC‐1 cells treated as indicated above. IP, immunoprecipitation; IB, immunobloting. Data shown are representative of three independent experiments

Inhibition of EGFR/VEGFR/IGF‐1R autophosphorylation by XQ‐2d‐His‐SH2 CM‐(Arg)9 was examined in both PANC‐1 and BxPC‐3 cells. These RTKs play antitumor role via multiple signaling cascades including PI3K/AKT, MAPK/ERK, and JAK/STAT pathways.^50^, ^51^, ^52^ The ability of XQ‐2d‐His‐SH2 CM‐(Arg)9 to block activation of pY‐related pathways was evaluated in PANC‐1 cells. The results unveiled that phosphorylation levels of EGFR, VEGFR2, IGF‐1R, Src, and STAT3 were decreased as the incubated time prolonged and the concentrations varied (Figure 5A). However, XQ‐2d‐His‐SH2 CM‐(Arg)9 augmented the phosphorylation of ERK1/2 and AKT, the downstream kinases of the EGFR signaling pathways,^52^ at different time points, which may be caused by other signaling pathways such as TGF‐β‐mediated signaling pathway.^53^ Furthermore, we investigate the long‐time effects on these RTKs, and the data showed the phosphorylation levels were attenuated after 6 h (Figure 5A). The results of BxPC‐3 and MiaPaca‐2 cells were consistent with PANC‐1 cells (Supporting information Figure S4A and S5A). These data indicated that XQ‐2d‐His‐SH2 CM‐(Arg)9 could be a potent inhibitor of phosphorylated protein‐mediated signaling pathways in cells such as EGFR,VEGFR, and IGF‐1R.

As XQ‐2d‐His‐SH2 CM‐(Arg)9 and His‐SH2 CM‐(Arg)9 exhibited similar pY‐binding and inhibitory ability in vitro as previously identified in Figure 2G, we utilized His‐SH2 CM‐(Arg)9 to investigate whether SH2 superbinder could block signal transduction in pY‐related pathways. Dysregulation in activity of EGFR, IGF‐1R, and VEGFR is highly correlated with a lot of cancers.^54^, ^55^, ^56^ The enhanced affinity between SH2 superbinder and the pY implied that they may function as an inhibitor of pY‐based signaling pathways. To test this hypothesis, we incubated the PANC‐1 WCLs with His‐SH2 CM‐(Arg)9, His‐SH2 Wt‐(Arg)9, and His‐(Arg)9, respectively. The data exhibited His‐(Arg)9 would not capture EGFR, and His‐SH2 Wt‐(Arg)9 bound less to EGFR compared with His‐SH2 CM‐(Arg)9 (Figure 5B). GRB2, a critical adaptor protein in EGFR signaling,^57^ bound substantially tighter to EGFR in His‐SH2 Wt‐(Arg)9 group than the His‐SH2 CM‐(Arg)9 (Figure 5B). Consistent with the EGFR/GRB2 interaction process, the downstream key proteins of IGF‐1R and VEGFR2,^58^, ^59^ such as IRS‐1 and SHC, bound less to IGF‐1R and VEGFR2, respectively (Figure 5C and D), in the His‐SH2 CM‐(Arg)9‐treated cell lysates, compared to the His‐SH2 Wt‐(Arg)9‐treated group. Similar results were observed in BxPC‐3 and MiaPaca‐2 cells (Supporting information Figure S4B to S4D, S5B to S5D). These data indicated that His‐SH2 CM‐(Arg)9 inhibited the EGFR/IGF‐1R/VEGFR2‐dependent pathways, likely by competing for capturing the pYs on the receptors.

### 3.5 XQ‐2d‐His‐SH2 CM‐(Arg)9 conjugate showed strong inhibitory effect on PSCs in pancreatic cancer stroma

The PDAC stroma was reported as the barrier to impede the delivery of drugs to the tumor cells.^60^ The first stroma‐targeting agents on clinical trial were SHH inhibitors. Although multiple trials continue to be ongoing, many have failed with striking discrepancy in results from preclinical data.^60^ How to break the stroma and improve the drug delivery remains the great challenge for PDAC therapy. The PDAC stroma mainly consists of activated PSCs and extracellular matrix (ECM) secreted by PSCs. Inactivating PSCs and decreasing the secretion of ECM was proved to be a good way to break the stroma. The expression of αSMA, one main marker of activated PSCs, had been evaluated in clinical specimens from 233 PDAC patients. Data from previous studies proved PSC activity was highly associated with clinical outcomes, indicating the importance and value of stroma in the therapy of PDAC.^61^, ^62^

**FIGURE 6:**
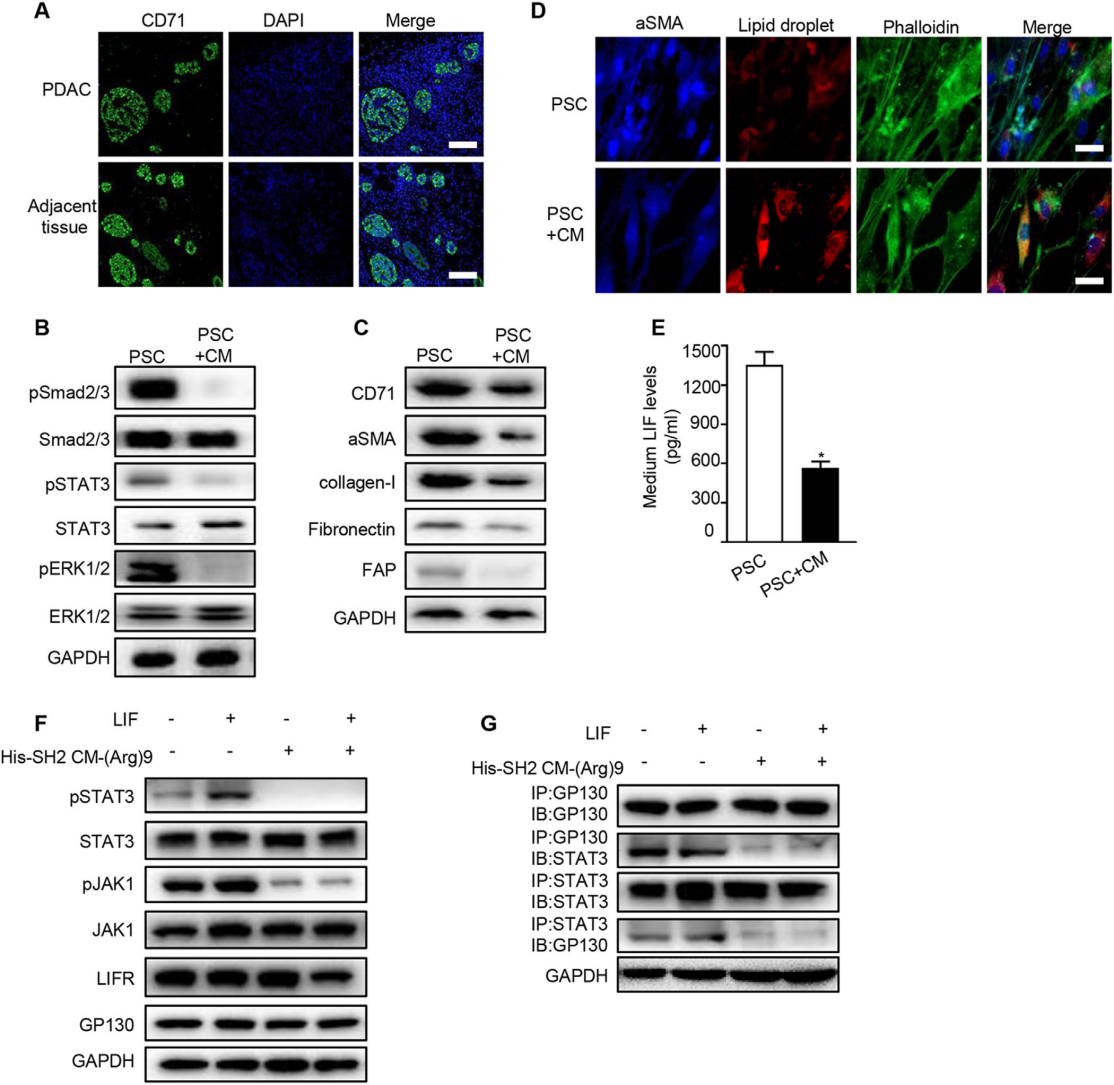
XQ‐2d‐His‐SH2 CM‐(Arg)9 showed inhibitory effect on PSCs in pancreatic cancer stroma. (A) IF staining images of tumor tissues and the adjacent tissues showing high CD71 expression. (B) XQ‐2d‐His‐SH2 CM‐(Arg)9 decreased phosphorylation levels of STAT3, Smad2/3, and ERK1/2 in active PSCs. (C) XQ‐2d‐His‐SH2 CM‐(Arg)9 reduced expression levels of αSMA, collagen‐I, FAP, and Fibronectin. (D) IF staining images of PSCs confirmed that XQ‐2d‐His‐SH2 CM‐(Arg)9 contributed to decreasing the levels of αSMA and inducing many lipid droplets (marker of quiescent PSCs). (E) XQ‐2d‐His‐SH2 CM‐(Arg)9 could decrease LIF in activated PSCs. (F) Effects of His‐SH2 CM‐(Arg)9 suppressing the phosphorylation caused by LIF in PANC‐1 cells. (G) Interaction of GP130 and STAT3 was affected by His‐SH2 CM‐(Arg)9. IP, immunoprecipitation; IB, immunobloting. “CM” in (B‐E) representing XQ‐2d‐His‐SH2 CM‐(Arg)9 conjugate. Data shown are representative of three independent experiments

The expression level of CD71 was detected in PDAC patient samples by immunohistochemical analysis, and both tumor tissue and the adjacent stroma showed high CD71 expression, which could be the target of XQ‐2d aptamer (Figure 6A). Experiments were carried out to evaluate whether XQ‐2d‐His‐SH2 CM‐(Arg)9 may recognize, capture, and inactivate PSCs. The results showed XQ‐2d‐His‐SH2 CM‐(Arg)9 conjugate could be delivered into desmoplastic stroma adjacent to the tumor site relying on the XQ‐2d aptamer. Next, we investigated whether XQ‐2d‐His‐SH2 CM‐(Arg)9 conjugate may revert PSCs from activation back to the inactive state. The results demonstrated that phosphorylation levels of STAT3, Smad2/3, and ERK1/2 were decreased by the conjugate, revealing the reduction of the activated phenotype (Figure 6B). Meanwhile, several major components of the pancreatic cancer stroma (αSMA, fibronectin, collagen‐I, and FAP) were significantly decreased (Figure 6C), implying that the ECM was modulated. By disrupting the activation of PSCs, the dense desmoplasia, one barrier of PDAC, was decimated by XQ‐2d‐His‐SH2 CM‐(Arg)9. The conjugate could penetrate stroma to tumor cells, further inhibiting tumor growth and metastasis. Furthermore, images of PSCs proved that XQ‐2d‐His‐SH2 CM‐(Arg)9 contributed to decreasing the level of αSMA (Figure 6D), which was in accordance with the results of Figure 6C. Meanwhile, images of vitamin A‐storing lipid droplets (marker of quiescent PSCs) revealed that XQ‐2d‐His‐SH2 CM‐(Arg)9 could effectively induce many lipid droplets in PSCs (Figure 6D). These data proved that XQ‐2d‐His‐SH2 CM‐(Arg)9 could decimate the dense stroma through disrupting the activation of PSCs and then attack the tumor cells.

Moreover, the LIF had been reported to be a key paracrine factor from activated PSCs acting on PDAC cells, which could be a potent target to PDAC therapy through blockade of the LIFR/GP130‐mediated signaling pathways.^63^ We further verified that His‐SH2 CM‐(Arg)9 could decrease LIF in activated PSCs (Figure 6E). The results revealed that His‐SH2 CM‐(Arg)9 could diminish the activation of JAK1 and STAT3 caused by LIF in PANC‐1 cells (Figure 6F). The results indicated that GP130 bound with less STAT3 after His‐SH2 CM‐(Arg)9 administration (Figure 6G). All these data proved that His‐SH2 CM‐(Arg)9 might block the LIFR/GP130 signaling pathway, probably by seizing the pY residues on LIFR, GP130, and STAT3.

### 3.6 XQ‐2d‐His‐SH2 CM‐(Arg)9 displayed targeting antitumor efficacy in vivo

**FIGURE 7:**
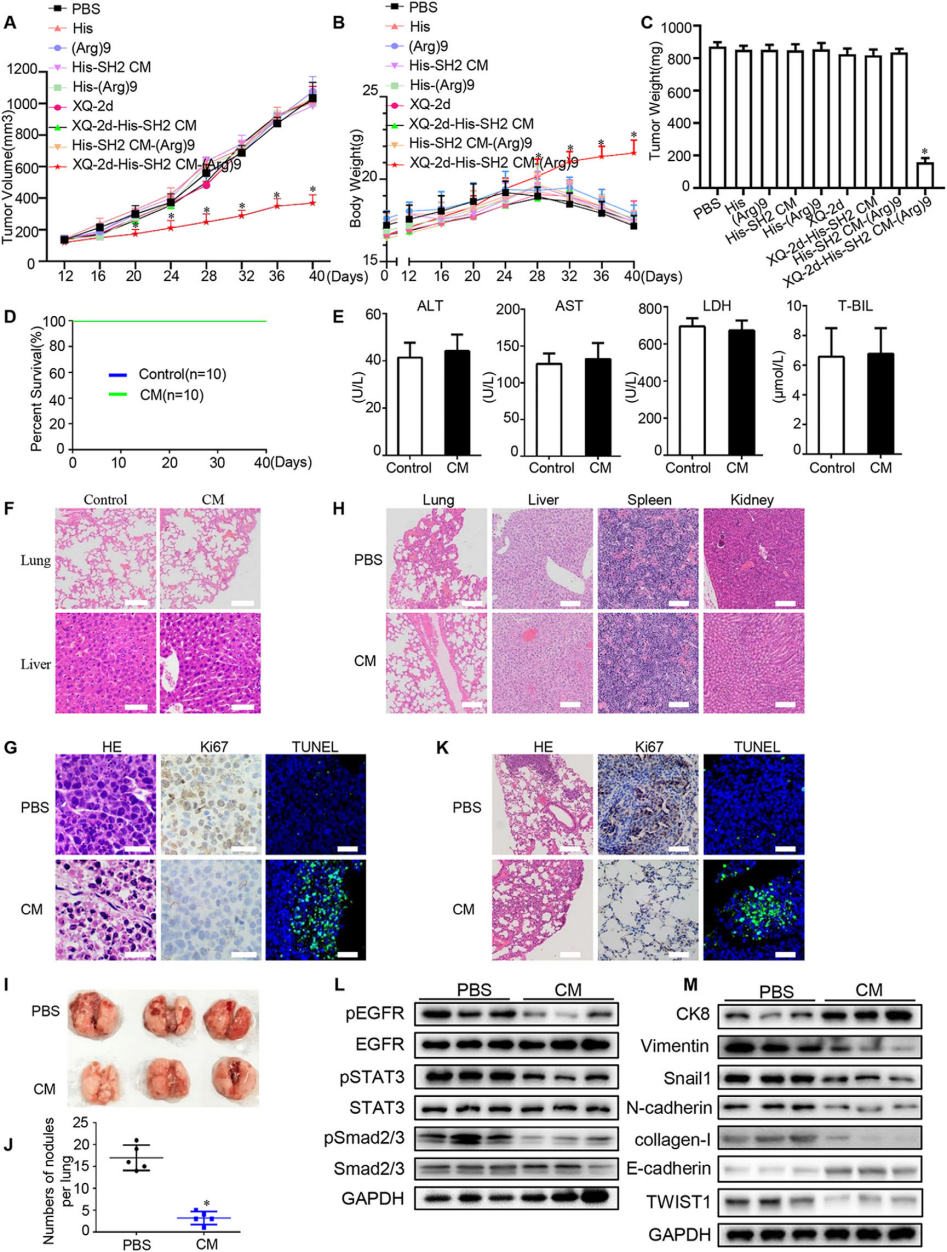
XQ‐2d‐His‐SH2 CM‐(Arg)9 displayed antitumor efficacy in vivo. (A) Effects of XQ‐2d‐His‐SH2 CM‐(Arg)9 on the growth of PANC‐1 tumors. The changes of tumor volumes were shown between XQ‐2d‐His‐SH2 CM‐(Arg)9 and PBS control (n = 10, *P < 0.05). (B) The body weight of mice in different groups. (C) The weight of dissected tumors. (D) The survival rate analysis showed that XQ‐2d‐His‐SH2 CM‐(Arg)9 could not hurt mice as PBS control (n = 10). (E) Hemanalysis was performed on mice at day 30 postdrug treatment. (F) HE staining of liver and lung from mice treated with XQ‐2d‐His‐SH2 CM‐(Arg)9 and PBS. Scale bar: 50 μm. (G) Representative histological examination of the dissected tumors using HE staining, TUNEL assay, and Ki67 staining. (H) HE staining of some organs from mice injected with XQ‐2d‐His‐SH2 CM‐(Arg)9 and PBS. Scale bar: 50 μm. (I, J) Mice were injected with 2 × 10^5^ PANC‐1 cells through vein tail and were sacrificed on day 14 postinjection. Images and quantification analysis of metastasis of mice injected with XQ‐2d‐His‐SH2 CM‐(Arg)9 or PBS. (K) Representative histological examination of the dissected lungs using HE staining, Ki67 staining, and TUNEL assay. (L) Western blot was used to identify whether XQ‐2d‐His‐SH2 CM‐(Arg)9 could regulate the phosphorylation levels of EGFR, STAT3, and Smad2/3 (n = 3). (M) EMT‐related markers were examined (n = 3). “CM” in (D‐M) was XQ‐2d‐His‐SH2 CM‐(Arg)9 conjugate. All images shown are representative of at least three independent experiments

**FIGURE 8:**
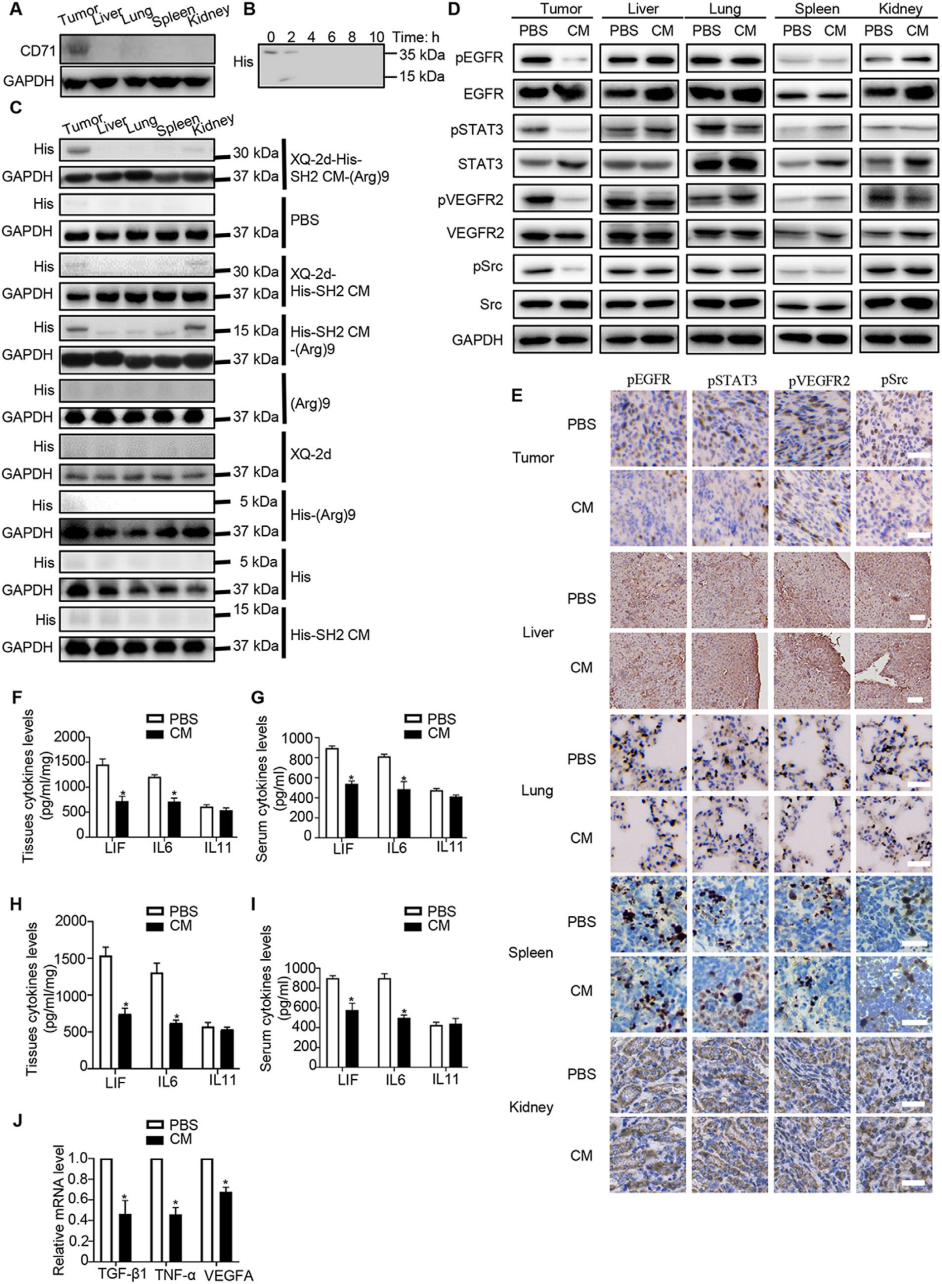
XQ‐2d‐His‐SH2 CM‐(Arg)9 showed targeting antitumor ability. (A) The expression levels of CD71 were detected in several tissues. (B) Degradation of XQ‐2d‐His‐SH2 CM‐(Arg)9 conjugate in tumors. (C) All his‐tagged proteins and XQ‐2d were injected into mice through tail vein. Western blot was used to examine their release and distribution. (D, E) Results of western blot and IHC assays. XQ‐2d‐His‐SH2 CM‐(Arg)9 could decrease the phosphorylation levels of EGFR, VEGFR2, STAT3, and Src in the tumors, whereas it had no effect on other tissues. Scale bar: 50 μm. (F, G) ELISA of LIF, IL6, and IL11 levels in tissues and serum in tumor‐bearing models (n = 3, *P < 0.05). (H, I) ELISA of LIF, IL6, and IL11 levels in tissues and serum in metastasis models (n = 3,*P < 0.05). (J) Relative mRNA levels of VEGF‐A, TGF‐β, and TNF‐α (n = 3, *P < 0.05). Data shown are representative of three independent experiments

To further confirm the role of XQ‐2d‐His‐SH2 CM‐(Arg)9 in vivo, tumor‐bearing mouse model was established. When the tumor size reached about 200‐300 mm^3^, mice were injected with different agents through tail vein every day. Tumor volumes of XQ‐2d‐His‐SH2 CM‐(Arg)9‐treated group were much smaller than those of other groups. The results demonstrated that XQ‐2d‐His‐SH2 CM‐(Arg)9 slowed down the growing rate of PANC‐1 tumors and increased body weight significantly compared to eight control groups (Figure 7A to C). Then, we investigate the expression levels of CD71 in tumors and other tissues, and the data showed that CD71 was highly expressed in tumors (Figure 8A).We further assessed drug degradation, release and distribution in tumors after injection via tail vein by western blot (Figure 8B and C). These results indicated that XQ‐2d‐His‐SH2 CM‐(Arg)9 could recognize and target the tumor tissues, further inhibiting tumor growth. Meanwhile, it would degrade at about 4 h in tumors.

Moreover, safety is an essential aspect for the development of effective translational therapy. To evaluate the biocompatibility of XQ‐2d‐His‐SH2 CM‐(Arg)9, hemanalysis was performed from the mice on day 30 postdrug treatment. The survival rate analysis showed XQ‐2d‐His‐SH2 CM‐(Arg)9 could not hurt mice as PBS group (Figure 7D). And both XQ‐2d‐His‐SH2 CM‐(Arg)9 and PBS‐treated mice showed normal levels of alanine aminotransferase (ALT), aspartate aminotransferase (AST), lactate dehydrogenase (LDH), and total bilirubin (TBIL) (Figure 7E). The histopathological analyses of lung and liver also showed in healthy condition (Figure 7F).

After confirming the strong antitumor effect and its safety profile of the conjugate in vivo, some main signaling pathways were investigated in several tissues. Consistent with the results in vitro, XQ‐2d‐His‐SH2 CM‐(Arg)9 could decrease the phosphorylated levels of EGFR, STAT3, VEGFR2, and Src in the tumors after XQ‐2d‐His‐SH2 CM‐(Arg)9 treatment, whereas it had no effect on other organs (Figure 8D). IHC assays of pEGFR, pVEGFR2, pSTAT3, and pSrc in tumors were conducted to strengthen the conclusions (Figure 8E). The data meant the conjugate could only penetrate into tumor tissues without harming healthy organs. These results were consistent with HE staining, Ki67 staining, and TUNEL assay, which showed that the conjugate resulted in higher level of necrotic lesions, lower level of proliferation ability, and higher apoptosis rate, compared to PBS group (Figure 7G). Histopathological examination of liver, lung, spleen, and kidney presented these tissues remained in healthy condition and would not be affected by the conjugate at the dose used in our study (Figure 7H).

**FIGURE 9:**
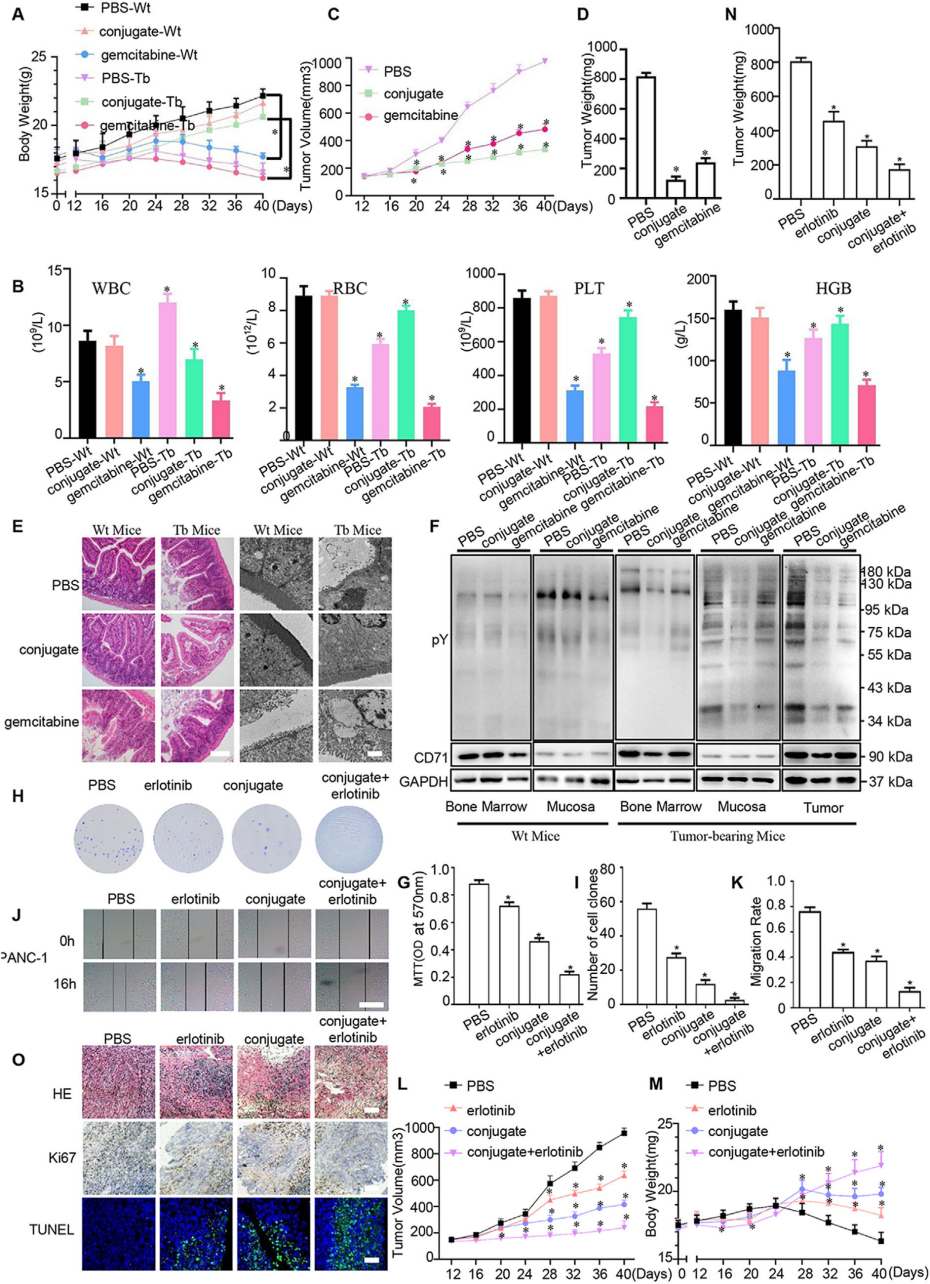
Comparison of gemcitabine, erlotinib, and XQ‐2d‐His‐SH2 CM‐(Arg)9 on antitumor efficacy and the side‐effects. (A) Effects of XQ‐2d‐His‐SH2 CM‐(Arg)9 and gemcitabine on the body weight of wild type and tumor‐bearing mice (n = 5, *P < 0.05). (B) Hemanalysis was performed on mice. (C) The tumor volume of tumor‐bearing mice in three different groups. (D) The weight of dissected tumors in tumor‐bearing mice. (E) HE staining of paraffin sections and representative electronic micrographs of small intestine tissues. Scale bar of HE: 50 μm. Scale bar of TEM: 2 μm. (F) Different pY and CD71 levels of bone marrows and small intestines were detected in wild type and tumor‐bearing mice. Mice were treated with PBS, gemcitabine (100 mg/kg i.p. twice a week), or conjugate (5 mg/kg) (n = 5, *P < 0.05). “Wt” was wild type; “Tb” was tumor‐bearing; “conjugate” was XQ‐2d‐His‐SH2 CM‐(Arg)9. (G) MTT assays were conducted to evaluate the effects of erlotinib and XQ‐2d‐His‐SH2 CM‐(Arg)9 on the proliferation of PANC‐1 cells. Cells were treated with PBS, erlotinib (24 μM, 24 h), conjugate (100 nM, 3 h), erlotinib + conjugate (n = 3, *P < 0.05). (H) Representative images of colony formation assays showing colonies formed by cells incubated with different administrations. (I) Bar graph depicting changes in number of cell colonies (n = 3, *P < 0.05). (J) Wound healing assays were monitored at 0 and 16 h in PANC‐1 cells with different agents. Scale bar: 100 μm. (K) Bar graph depicting changes in migration rate (n = 3, *P < 0.05). (L) Effects of erlotinib (25 mg/kg) and conjugate (5 mg/kg) on the growth of PANC‐1 tumors (n = 5, *P < 0.05). (M) The body weight of mice in different groups. (N) The weight of dissected tumors. (O) Representative histological examination of the dissected tumors using HE staining, TUNEL assay, and Ki67 staining. Scale bar: 50 μm. All images shown are representative of at least three independent experiments

Clinically, bone marrow inhibition and mucosa damage are main problems of conventional antitumor drugs.^64^, ^65^ Given that gemcitabine is a widely used drug in the therapy of pancreatic cancer,^66^ it was set as a control to evaluate the side‐effects of the conjugate. In Wt mice, mice in PBS and conjugate (5 mg/kg) groups showed normal growth of body weights, whereas mice in gemcitabine (50 mg/kg) group appeared significant weight loss (Figure 9A). To evaluate the bone marrow inhibition, hemanalysis was performed with blood from mice when they were sacrificed. The PBS‐ or conjugate‐treated groups showed similar levels of red and white blood cell (RBC and WBC) number, hemoglobin (HGB), platelet (PLT) number, monocyte, lymphocyte, and neutrophil granulocyte number, and other indexes; whereas gemcitabine could obviously affect them (Figure 9B, Supporting information Table S4). In tumor‐bearing mice, mice in PBS‐ and gemcitabine‐treated groups appeared major weight loss (Figure 9A). The conjugate made tumors smaller and lighter than gemcitabine (Figure 9C and D). Mice in PBS group had abnormal levels of WBC, RBC, PLT, HGB, possibly due to the larger tumor burden (Figure 9B, Supporting information Table S4). Compared to gemcitabine, these parameters remained nearly normal under conjugate administration, meaning that the conjugate could exhibit better therapy efficacy and cause less side‐effect than gemcitabine (Figure 9B). We further examined the morphology of intestinal mucosa by H&E staining and Transmission electron microscopy (TEM). Compared with PBS or the conjugate, gemcitabine obviously destroyed the integrity of intestinal mucosa (Figure 9E). Moreover, the levels of pY and CD71 were detected in tumor, bone marrow, and mucosa (Figure 9F). The data demonstrated that the conjugate would not affect CD71 or pY levels in Wt mice and could slightly hurt CD71 and significantly decrease pY levels of tumor‐bearing mice. Briefly, during the entire process of mouse‐modelling, compared to PBS‐ or conjugate‐treated groups, mice of gemcitabine administration suffered from lack of appetite, hair loss, and so on. For Wt mice, the results showed the conjugate caused negligible side‐effect on bone marrow and mucosa compared with gemcitabine. For tumor‐bearing mice, the results displayed that the conjugate showed slight side‐effect on bone marrow and mucosa compared with gemcitabine.

Interleukin6 (IL‐6) and other interleukin family members (eg, LIF) bind to GP130‐containing receptor complexes to activate similar signaling cascades in PDAC, such as STAT3 mediated pathway.^63^ The results showed that XQ‐2d‐His‐SH2 CM‐(Arg)9 could decrease the concentrations of IL6 and LIF in PDAC mouse model in the tumor tissues and blood, whereas IL‐11 exhibited no obvious change (Figure 8F and G). All above‐mentioned findings underscored the importance of regulation of XQ‐2d‐His‐SH2 CM‐(Arg)9 to IL6‐ mediated signaling in PDAC.

To investigate whether XQ‐2d‐His‐SH2 CM‐(Arg)9 would play a role in tumor metastasis, we inoculated nude mice with PANC‐1 cells intravenously and inspected lungs for analysis. XQ‐2d‐His‐SH2 CM‐(Arg)9‐treated group exhibited significantly less tumor nodules compared to the PBS control group (Figure 7I and J). Histologically, the metastatic areas, amounts of Ki67, and apoptosis rate, in lungs were statistically lower following XQ‐2d‐His‐SH2 CM‐(Arg)9 administration compared with the control groups (Figure 7K). In addition, pSmad2/3, pEGFR and pSTAT3 were decreased, indicating that TGF‐β and EGFR ‐mediated signaling pathways were downregulated by XQ‐2d‐His‐SH2 CM‐(Arg)9 (Figure 7L). The data indicated CK8 was increased, whereas *N*‐cadherin, Snail1, collagen‐I, and Vimentin decreased in the tumors of XQ‐2d‐His‐SH2 CM‐(Arg)9‐treated mice compared to PBS control group (Figure 7M). As SH2 superbinder has been verified to block the IL6R‐mediated signaling pathway, we detected the levels of several cellular markers. The data revealed that XQ‐2d‐His‐SH2 CM‐(Arg)9 could reduce the concentrations of IL6 and LIF in PDAC mouse model in the tumor tissues and blood, whereas IL11 showed no obvious change (Figure 8H and I).

Previous studies have described that epithelial and carcinoma cells would lose their epithelial characteristics, while cancer‐associated fibroblasts (CAFs)‐mediated pathways were activated, including TGF‐β, TNF‐α, and vascular endothelial growth factor (VEGF).^67^, ^68^, ^69^ TGF‐β acts on endothelial cell proliferation, migration and capillary formation, and thereby promoting metastasis and angiogenesis. A major factor for vascularization is VEGF, which is induced by TGF‐β.^70^ In common with other family members, TNF‐α is involved in maintenance and homeostasis of the immune system, inflammation, and host defense.^71^ Accordance with previous findings, XQ‐2d‐His‐SH2 CM‐(Arg)9 impeded mRNA expression levels of TGF‐β, TNF‐α, and VEGF‐A (Figure 8J). Taken together, these data demonstrated that XQ‐2d‐His‐SH2 CM‐(Arg)9 could suppress tumor metastasis in a lung metastasis model.

## 4 DISCUSSION

Aberrant activation of tyrosine kinases plays a vital role in cancer.^9^ Many tumor‐targeted drugs have been used in many kinds of cancers; however, they have failed to show obvious effect to improve patient survival of pancreatic cancer.^4^ The main reasons probably lie in two aspects. The first reason is that dense stroma obstructs the delivery of drugs to the tumor cells. The other reason may be attributed to the compensatory activation of the sophisticated signaling pathways in tumor cells.^21^, ^60^

In our study, we found activated PSCs, key component of the PDAC stroma, also showed high CD71 protein expression, which could be the target of XQ‐2d. Thus, XQ‐2d‐His‐SH2 CM‐(Arg)9 could specifically bind and then be translocated into the PSCs. Furthermore, XQ‐2d‐His‐SH2 CM‐(Arg)9 conjugate could revert active PSCs back to the inactive state and decreased the secretion of ECM by PSCs through downregulating the phosphorylation levels of STAT3, Smad2/3, and ERK1/2. In this way, XQ‐2d‐His‐SH2 CM‐(Arg)9 conjugate may eradicate the dense PDAC stroma and facilitate the delivery of the conjugate to the tumor cells.

Conventional tumor‐targeted drugs are usually against one or two kinases, which is hard to block the sophisticated signaling cascades, so the effect is very weak in PDAC.^9^, ^12^ SH2 superbinder may be used as the broad‐spectrum inhibitor by blocking multitude pY‐based signaling pathways. Based on this advantage, XQ‐2d‐His‐SH2 CM‐(Arg)9 conjugate was verified to have potent antitumor efficacy in vitro and in vivo. Many phosphorylation signaling cascades regulating the proliferation and metastasis were inhibited by the conjugate, such as PI3K/AKT, MAPK/ERK and JAK/STAT pathways, under EGFR/VEGFR/IGF‐1R.

Benefiting from the ability of aptamer XQ‐2d, the conjugate may specifically recognize and enter into PDAC cells. By preventing the drugs from entering into the normal tissues and cells, XQ‐2d‐His‐SH2 CM‐(Arg)9 conjugate may dramatically decrease the concentration of drug administration and the side‐effect. In our previous research,^27^ the optimal concentration of (Arg)_9_‐GST SH2 superbinder protein was 1 μM. In contrast, it is only 100 nM for XQ‐2d‐His‐SH2 CM‐(Arg)9 conjugate in this study, which shows tenfold reduction compared with the former. Meanwhile, the conjugate showed slight or negligible side‐effect on bone marrow and mucosa in Wt or tumor‐bearing mice. In our opinion, the mechanisms mainly lie in two aspects. First, the molecular weight of the conjugate (XQ‐2d aptamer and SH2 protein) is about 26 KD, which is too huge to easily penetrate through capillary vessel and reach the normal organs or tissues, such as bone marrow and mucosa. As for tumor tissues, the conjugate is prone to reach the tumor sites because of the enhanced permeability and retention effect (EPR effect).^72^ Second, the tyrosine phosphorylation is dynamic in cells. The total pY level of bone marrow or intestinal mucosa is very low or moderate according to the data (Figure 9F). SH2 protein might gradually degrade and lose its function to block pY‐related signaling in cells. Thus, even if the conjugate might enter into bone marrow and mucosa, it is hard to do harm to them.

XQ‐2d‐His‐SH2 CM‐(Arg)9 conjugate also impaired the ability of pancreatic cancer cells to undergo EMT by upregulating the expression of CK8 and downregulating Vimentin, Snail and *N*‐cadherin. Many cytokines secreted by the PSCs or tumor cells contribute to the development of PDAC such as LIF.^63^ SH2 superbinder has been verified to block the LIFR/STAT3 pathway and interrupt the tumor progression. As a candidate drug, safety is an essential prerequisite for it. XQ‐2d‐His‐SH2 CM‐(Arg)9 conjugate exhibits excellent safety according to the hemanalysis and survival rate analysis of mice.

**FIGURE 10:**
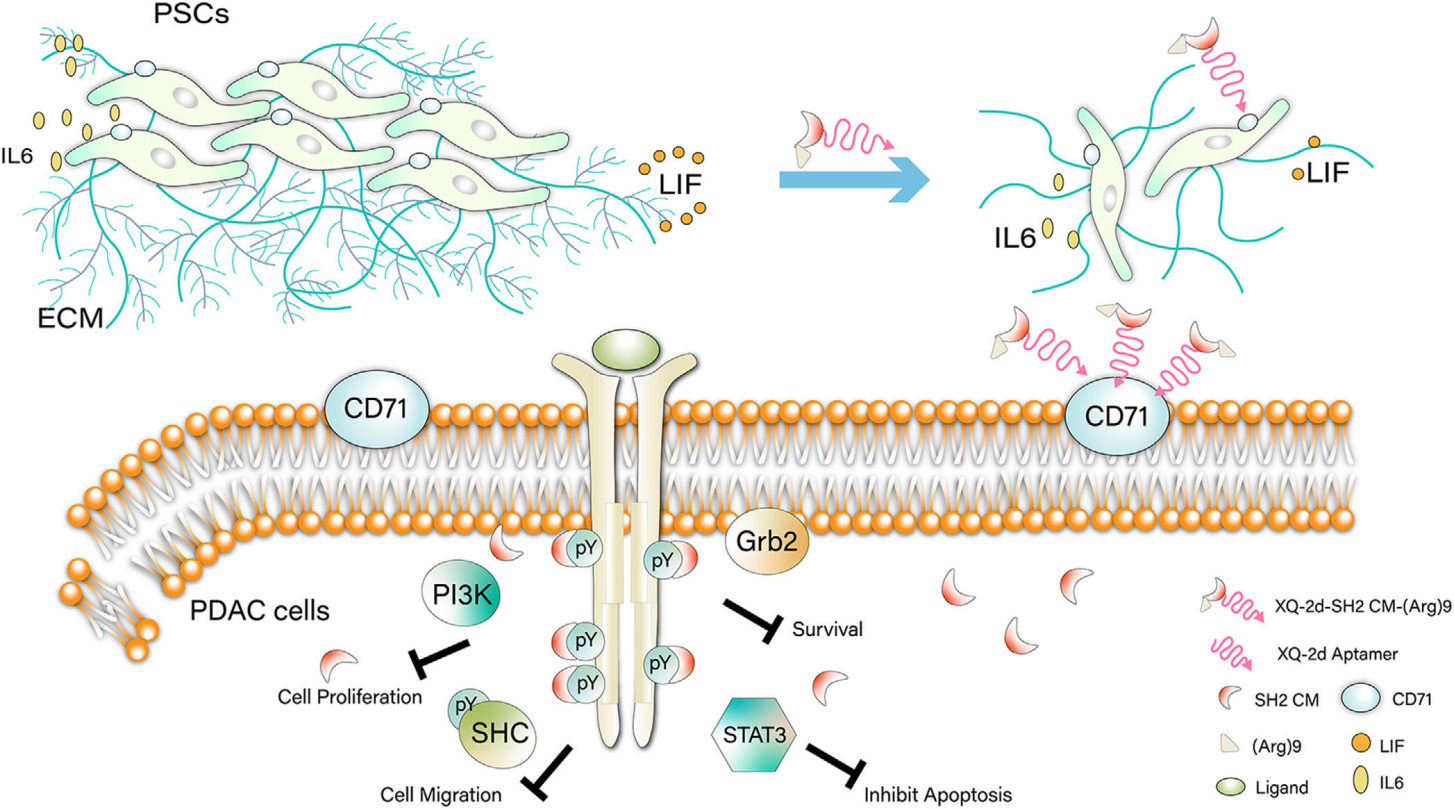
Schematic diagram of XQ‐2d‐His‐SH2 CM‐(Arg)9 influencing PDAC cells and PSCs. XQ‐2d‐His‐SH2 CM‐(Arg)9 could specifically bind and then be translocated into PSCs. Furthermore, XQ‐2d‐His‐SH2 CM‐(Arg)9 could revert activated PSCs back to the inactive state and decrease the secretion of ECM. After eliminating the dense stroma, XQ‐2d‐His‐SH2 CM‐(Arg)9 could arrive at the tumor tissues. Then it would recognize and enter into the PDAC cells, capturing pY residues of proteins to function as a broad‐spectrum inhibitor via blocking multitude pY‐based signaling pathways

Taken together, PDAC tumor tissues and cell lines exhibited high pY levels, which has an essential role in PDAC carcinogenesis. XQ‐2d‐His‐SH2 CM‐(Arg)9 could eliminate the dense stroma of PDAC and then arrive at the tumor tissues. XQ‐2d‐His‐SH2 CM‐(Arg)9 could capture the pY residues of proteins to function as a broad‐spectrum inhibitor for blocking multitude pY‐based signaling pathways. Our results indicated that XQ‐2d‐His‐SH2 CM‐(Arg)9 could inhibit proliferation, invasion, and metastasis of PDAC cells with potent efficacy via blocking the activities of several pY‐related signaling cascades (Figure 10). Considering the extreme complexity of the signal transduction in cells, it is hard to elaborate the specific mechanism of the SH2 superbinder to inhibit multiple, not all, RTKs and CTKs in our studies.

Erlotinib, a widely used EGFR TKI for cancer therapy with proven efficacy, has been investigated in a lot of cancers, such as non‐small cell lung cancer (NSCLC) and PDAC.^73^, ^74^ To compare the therapy efficacy of XQ‐2d‐His‐SH2 CM‐(Arg)9 and erlotinib, several experiments were conducted. The administration of erlotinib (Targetmol, Shanghai) referred to previous study.^75^ The results displayed that XQ‐2d‐His‐SH2 CM‐(Arg)9 elicited better inhibitory effect than erlotinib in vitro and in vivo (Figure 9G and O), maybe due to the multitarget characteristic of XQ‐2d‐His‐SH2 CM‐(Arg)9. And combination therapy with XQ‐2d‐His‐SH2 CM‐(Arg)9 and erlotinib showed more efficacious, which might be considered in the future application.

This novel strategy, Aptamer‐SH2 superbinder‐(Arg)9, also provides a universal targeted avenue to devastate cancer cells by using distinctive DNA aptamer against different cancers. Precision guide of aptamer prevents the drugs from entering into the normal tissues and cells, by which it may decrease the side‐effect to the minimum and contribute to high drug concentration around the tumor tissues and cells. SH2 superbinder may block multiple pY‐based signaling pathways in tumor cells. Of course, several aspects need to be improved for our current conjugate. The preparation efficiency of the conjugate need to be optimized. In order to prolong half‐life of the conjugate in vivo, liposome technology is under consideration and the related research is ongoing. For further clinical cancer therapy, the Aptamer‐SH2 superbinder‐(Arg)9 can also be combined with TKI (eg, Erlotinib) or radiotherapy to promote the therapeutic effect. Hence, next work will concentrate on exploring better application of Aptamer‐SH2 superbinder‐(Arg)9 conjugate system in future.

## 5 CONCLUSIONS

PDAC tumor tissues and cell lines exhibited high pY levels, which has an essential role in PDAC carcinogenesis. The conjugate of XQ‐2d‐His‐SH2 CM‐(Arg)9 could recognize and enter into the PDAC cells and dense stroma, capturing the pY residues of proteins to function as a broad‐spectrum inhibitor for blocking multitude pY‐based signaling pathways. The results indicated that the conjugate could inhibit proliferation, invasion, and metastasis of PDAC cells with potent efficacy via regulating the activity of several pY‐related signaling cascades. The conjugate could eliminate the dense stroma of PDAC and then arrive at the tumor tissues.

## ETHICS APPROVAL AND CONSENT TO PARTICIPATE

Tissue acquisition and handling of human tissue specimens in this study have been approved by the Ethics Committee of Tongji Medical College, Huazhong University of Science and Technology. All studies involving animals were performed following the National Guides for the Care and Use of Laboratory Animals and approved by the Institutional Animal Care and Use Committee of Tongji Medical College, Huazhong University of Science and Technology.

## AVAILABILITY OF DATA AND MATERIALS

The dataset supporting the conclusions of this article is included within the article and its additional file.

## CONFLICT OF INTEREST

The authors declare that they have no conflict of interests.

## AUTHOR CONTRIBUTIONS

ADL, JZ, and XYB conducted the experiments; GQH, QC, and HX participated in the data analysis. XC, ADL, and JZ designed the experiments and drafted the manuscript. SSCL polished the manuscript and gave some useful advice. All authors read and approved the final manuscript.

## FUNDING

This work was supported by the National Natural Science Foundation of China (Nos. 81974468, 31771541); the China Postdoctoral Science Foundation (No. 2018M642815); the Natural Science Foundation of Hubei Province of China (No. 2019CFB734); and the Hubei Postdoctoral Preferential Foundation (No. Z11).

## Supporting information


**Figure S1. Construction, expression and purification of His‐SH2 Wt‐(Arg)9, His‐SH2 TrM‐(Arg)9 and His‐SH2 CM‐(Arg)9**. (A) Schematic diagram of pETM11‐His‐SH2 Wt‐(Arg)9, pETM11‐His‐SH2 TrM‐(Arg)9 and pETM11‐His‐SH2 CM‐(Arg)9. (B) SDS‐PAGE Coomassie blue‐staining image displaying the expression and purification of His‐SH2 t‐(Arg)9, His‐SH2 TrM‐(Arg)9 and His‐SH2 CM‐(Arg)9 in *E.coli* BL21. Data shown are representative of three independent experiments.

**Figure S2. XQ‐2d‐SH2 CM‐(Arg)9 could not penetrate into some normal cell line or cells with low CD71 levels at a low concentration**. For normal cell line hTERT‐HPNE (A), only very weak signal of the conjugate at 250 nM was observed and no any signal at 100 nM and below when the incubation time was 3 h. (B) Under the same incubation condition, the conjugate could recognize cancer cells with high CD71 levels on the surface, such as H1299 and MDA‐MB‐231 cells; it failed to enter into cancer cells with low CD71 levels, such as MCF7. Cells were stained with anti‐His antibody followed by goat‐anti‐rabbit FITC secondary antibody incubation. Actin was stained with Rhodamine‐phallodin (Red) and nucleus with DAPI (Blue). Scale bar: 20 μm. All images shown are representative of at least three independent experiments.

**Figure S3. XQ‐2d‐His‐SH2 CM‐(Arg)9 exhibited obvious antitumor efficacy in MiaPaca‐2 cells and had no cytotoxity on hTERT‐HPNE or HPDE cells**. PANC‐1 cells were treated with XQ‐2d‐His‐SH2 CM‐(Arg)9 for different concentrations (A) or different time periods (B) to assess the inhibitory effect. (C) Effects of XQ‐2d‐His‐SH2 CM‐(Arg)9 on the proliferation of MiaPaca‐2 cells. Cells were treated with His‐alone, (Arg)9, His‐SH2 CM, His‐(Arg)9, XQ‐2d, XQ‐2d‐His‐SH2 CM, XQ‐2d‐His‐SH2 CM‐(Arg)9 and His‐SH2 CM‐(Arg)9 at 100 nM for 3 h. (D) Representative images of colony formation assay showing colonies formed by cells incubated with different agents. (E) Bar graph depicting changes in number of cell colonies. (F) Effects of XQ‐2d‐His‐SH2 CM‐(Arg)9 on the proliferation of hTERT‐HPNE and HPDE cells. (G) Representative images of colony formation assays showing colonies formed by cells incubated with different agents. (H) Bar graph depicting changes in number of cell colonies. (I) Wound healing assays were monitored at 0 h and 16 h in MiaPaca‐2 cells with different agents. Scale bar: 100 μm. (J) Bar graph depicting changes in migration rate. (K) Representative images and results of transwell assays of MiaPaca‐2 cells treated with different treatments. Scale bar: 20 μm. (L) Bar graph depicting changes of invasion rate. All images shown are representative of at least three independent experiments (*P < .05).

**Figure S4. XQ‐2d‐His‐SH2 CM‐(Arg)9 influenced multiple signaling cascades of BxPC‐3 cells**. (A) Inhibition of EGFR, VEGFR2, IGF‐1R, Src, AKT and ERK1/2 phosphorylation by XQ‐2d‐His‐SH2 CM‐(Arg)9 was examined in BxPC‐3 cells. (B‐D) The panels showed reciprocal immunoprecipitation of EGFR and GRB2 (B), IGF‐1R and IRS1(C), VEGFR2 and SHC (D) in BxPC‐3 cells treated as indicated above. IP, immunoprecipitation; IB, immunobloting. Data shown are representative of three independent experiments.

**Figure S5. XQ‐2d‐His‐SH2 CM‐(Arg)9 influenced multiple signaling cascades of MiaPaca‐2 cells**. (A) Inhibition of EGFR, VEGFR2, IGF‐1R, Src, AKT and ERK1/2 phosphorylation by XQ‐2d‐His‐SH2 CM‐(Arg)9 was examined in MiaPaca‐2 cells. (B‐D) The panels showed reciprocal immunoprecipitation of EGFR and GRB2 (B), IGF‐1R and IRS1(C), VEGFR2 and SHC (D) in MiaPaca‐2 cells treated as indicated above. IP, immunoprecipitation; IB, immunobloting. Data shown are representative of three independent experiments.

**Table S1**. Amino acid sequences of Src SH2 domain related variants. Src SH2 TrM contains three amino acid substitutions: T65V, C70A and K88L, shown in bold and red; Src SH2 CM domain contains four amino acid substitutions: T65V, C70A, K88L and C123S, shown in bold and red. Sequence of (Arg)9 is marked in green and bold. Sequence of 6 × His is marked in blue and bold. The recombinant protein was constructed based on the His‐tagged Src SH2 domain triple mutant protein with (Arg)9 at C‐terminus, named His‐SH2 TrM‐(Arg)9. Cysteine residue on site 123 of His‐SH2 TrM‐(Arg)9 was mutated to Serine without affecting the structure and binding property, and the modified SH2 superbinder was named SH2 CM.

**Table S2**. Primer sequences for recombinant plasmids. (Arg)9 was subcloned into pETM11‐SH2 Wt and pETM11‐SH2 TrM by using the One Step Cloning Kit. pETM11‐SH2 CM‐(Arg)9 was constructed by Fast Site‐Directed Mutagenesis Kit.

**Table S3**. Primer sequences for qPCR. Relative mRNA levels of CD71, VEGF‐A, TGFβ and TNFα were evaluated by qPCR. The Ct value for each sample was normalized based on the control of GAPDH gene.

**Table S4**. Hemanalysis was performed on mice when they were sacrificed.

**Dataset 1**. The levels of phosphotyrosine in multiple proteins were significantly elevated in pancreatic cancer cell lines. The excel file is shown in additional attachment.
