# Supplementary figures and images for: Aptamer‐SH2 superbinder‐based targeted therapy for pancreatic ductal adenocarcinoma
Source: Clin Transl Med. 2021 Feb 26;11(3):e337. doi: 10.1002/ctm2.337 (PMC7908048; doi:10.1002/ctm2.337)

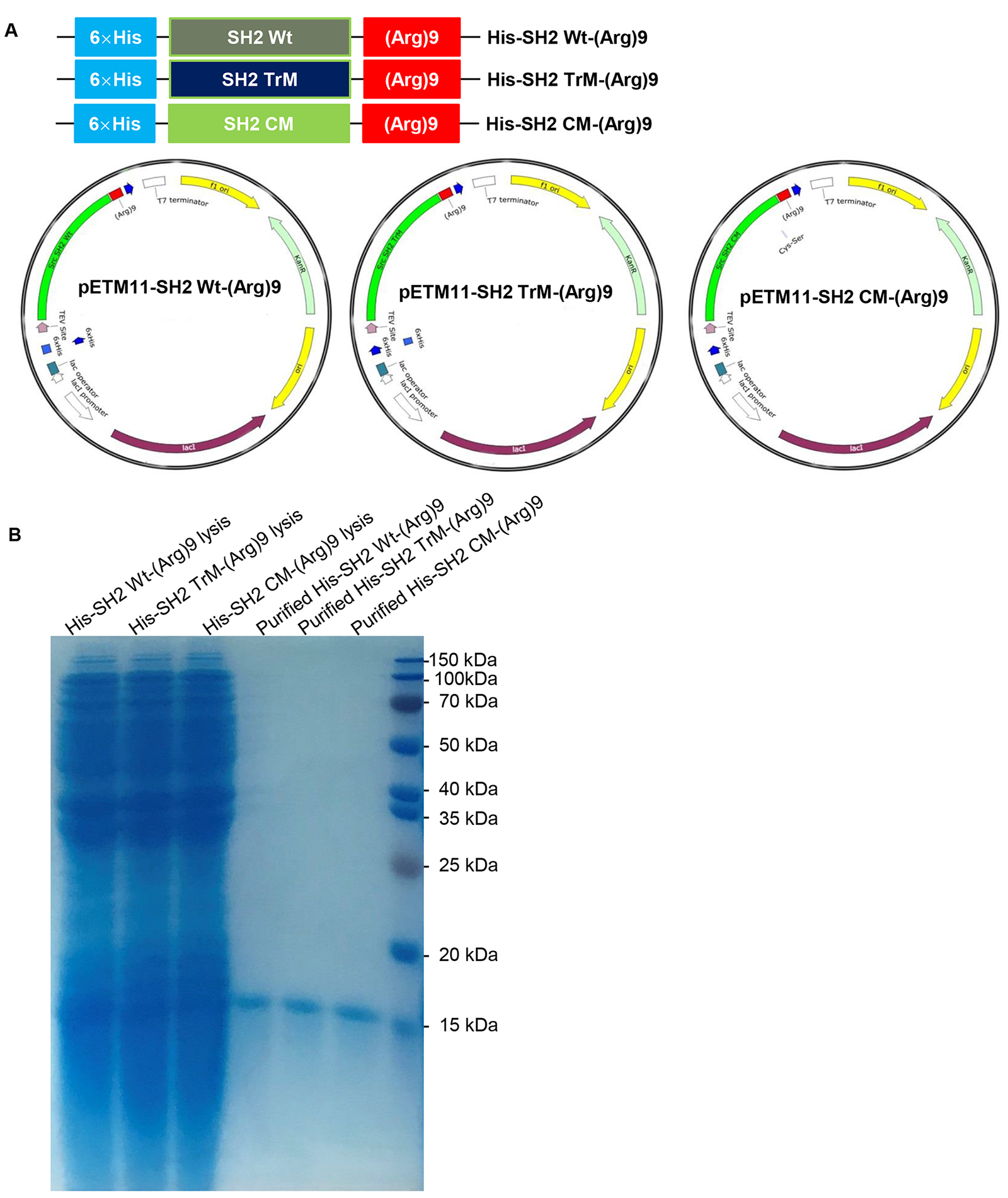

Supplement: Supplementary file 1 — Figure S1. Construction, expression and purification of His‐SH2 Wt‐(Arg)9, His‐SH2 TrM‐(Arg)9 and His‐SH2 CM‐(Arg)9. (A) Schematic diagram of pETM11‐His‐SH2 Wt‐(Arg)9, pETM11‐His‐SH2 TrM‐(Arg)9 and pETM11‐His‐SH2 CM‐(Arg)9. (B) SDS‐PAGE Coomassie blue‐staining image displaying the expression and purification of His‐SH2 t‐(Arg)9, His‐SH2 TrM‐(Arg)9 and His‐SH2 CM‐(Arg)9 in E.coli BL21. Data shown are representative of three independent experiments. [file CTM2-11-e337-s001.tif]

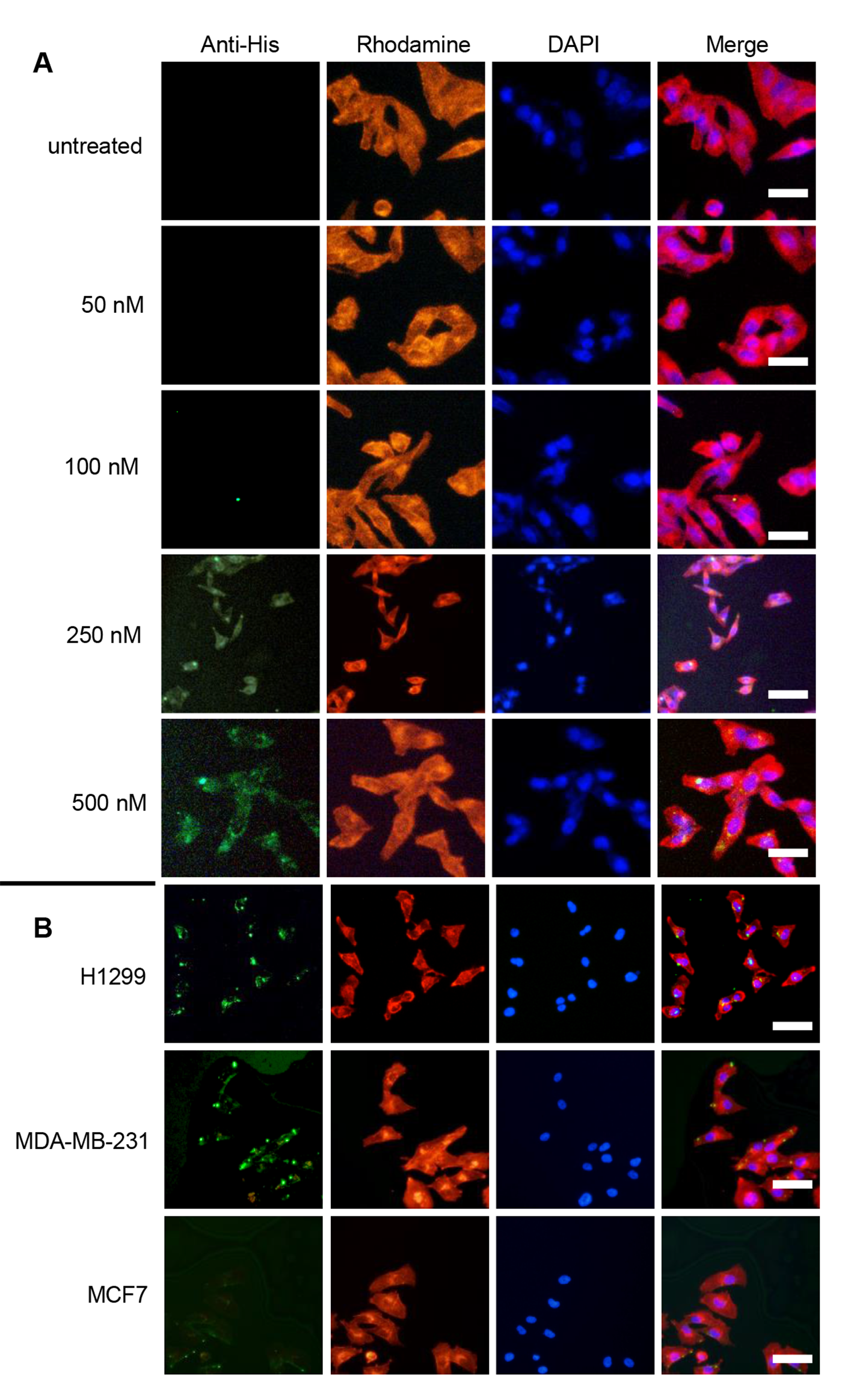

Supplement: Supplementary file 2 — Figure S2. XQ‐2d‐SH2 CM‐(Arg)9 could not penetrate into some normal cell line or cells with low CD71 levels at a low concentration. For normal cell line hTERT‐HPNE (A), only very weak signal of the conjugate at 250 nM was observed and no any signal at 100 nM and below when the incubation time was 3 h. (B) Under the same incubation condition, the conjugate could recognize cancer cells with high CD71 levels on the surface, such as H1299 and MDA‐MB‐231 cells; it failed to enter into cancer cells with low CD71 levels, such as MCF7. Cells were stained with anti‐His antibody followed by goat‐anti‐rabbit FITC secondary antibody incubation. Actin was stained with Rhodamine‐phallodin (Red) and nucleus with DAPI (Blue). Scale bar: 20 μm. All images shown are representative of at least three independent experiments. [file CTM2-11-e337-s002.tif]

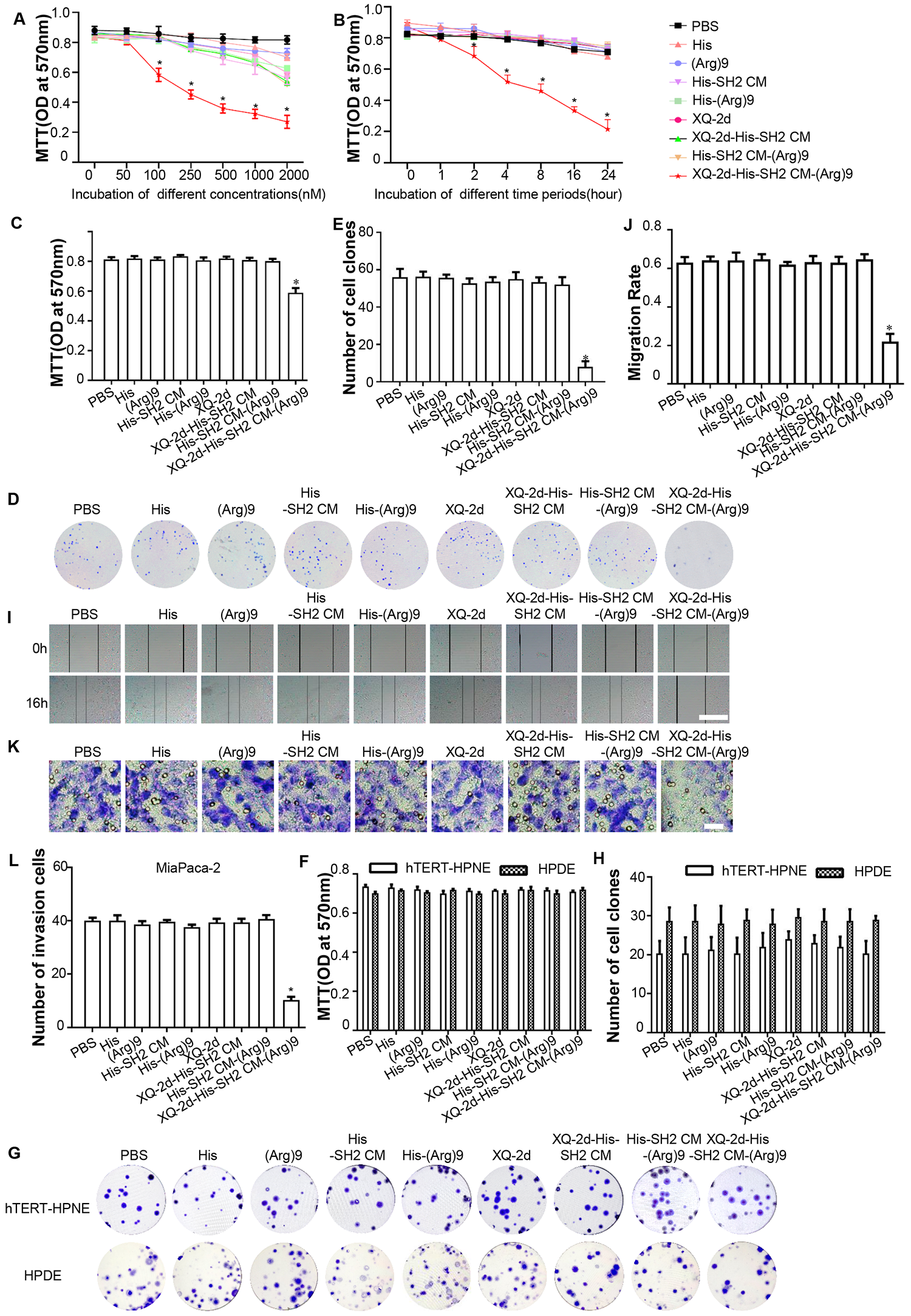

Supplement: Supplementary file 3 — Figure S3. XQ‐2d‐His‐SH2 CM‐(Arg)9 exhibited obvious antitumor efficacy in MiaPaca‐2 cells and had no cytotoxity on hTERT‐HPNE or HPDE cells. PANC‐1 cells were treated with XQ‐2d‐His‐SH2 CM‐(Arg)9 for different concentrations (A) or different time periods (B) to assess the inhibitory effect. (C) Effects of XQ‐2d‐His‐SH2 CM‐(Arg)9 on the proliferation of MiaPaca‐2 cells. Cells were treated with His‐alone, (Arg)9, His‐SH2 CM, His‐(Arg)9, XQ‐2d, XQ‐2d‐His‐SH2 CM, XQ‐2d‐His‐SH2 CM‐(Arg)9 and His‐SH2 CM‐(Arg)9 at 100 nM for 3 h. (D) Representative images of colony formation assay showing colonies formed by cells incubated with different agents. (E) Bar graph depicting changes in number of cell colonies. (F) Effects of XQ‐2d‐His‐SH2 CM‐(Arg)9 on the proliferation of hTERT‐HPNE and HPDE cells. (G) Representative images of colony formation assays showing colonies formed by cells incubated with different agents. (H) Bar graph depicting changes in number of cell colonies. (I) Wound healing assays were monitored at 0 h and 16 h in MiaPaca‐2 cells with different agents. Scale bar: 100 μm. (J) Bar graph depicting changes in migration rate. (K) Representative images and results of transwell assays of MiaPaca‐2 cells treated with different treatments. Scale bar: 20 μm. (L) Bar graph depicting changes of invasion rate. All images shown are representative of at least three independent experiments (*P < .05). [file CTM2-11-e337-s003.tif]

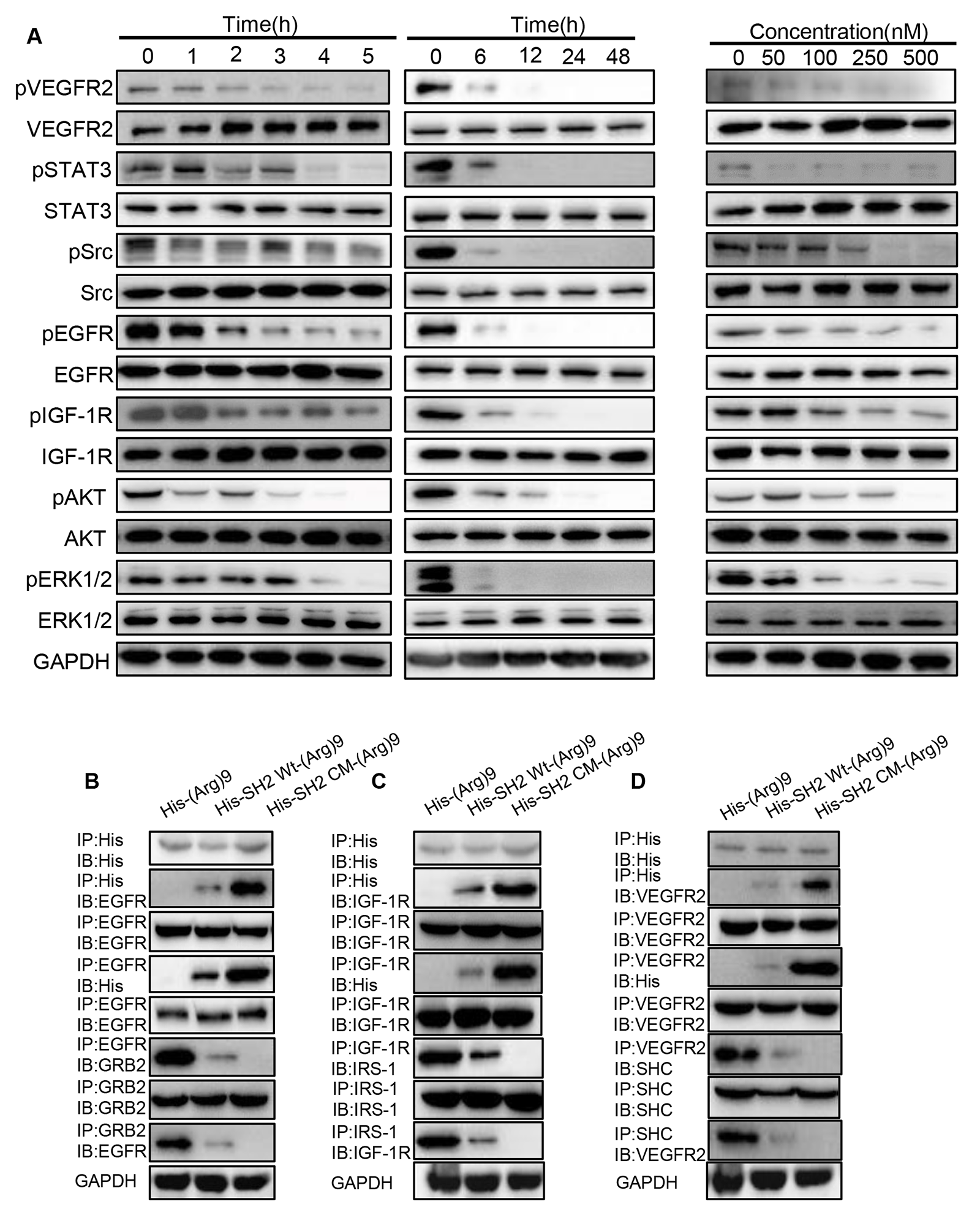

Supplement: Supplementary file 4 — Figure S4. XQ‐2d‐His‐SH2 CM‐(Arg)9 influenced multiple signaling cascades of BxPC‐3 cells. (A) Inhibition of EGFR, VEGFR2, IGF‐1R, Src, AKT and ERK1/2 phosphorylation by XQ‐2d‐His‐SH2 CM‐(Arg)9 was examined in BxPC‐3 cells. (B‐D) The panels showed reciprocal immunoprecipitation of EGFR and GRB2 (B), IGF‐1R and IRS1(C), VEGFR2 and SHC (D) in BxPC‐3 cells treated as indicated above. IP, immunoprecipitation; IB, immunobloting. Data shown are representative of three independent experiments. [file CTM2-11-e337-s004.tif]

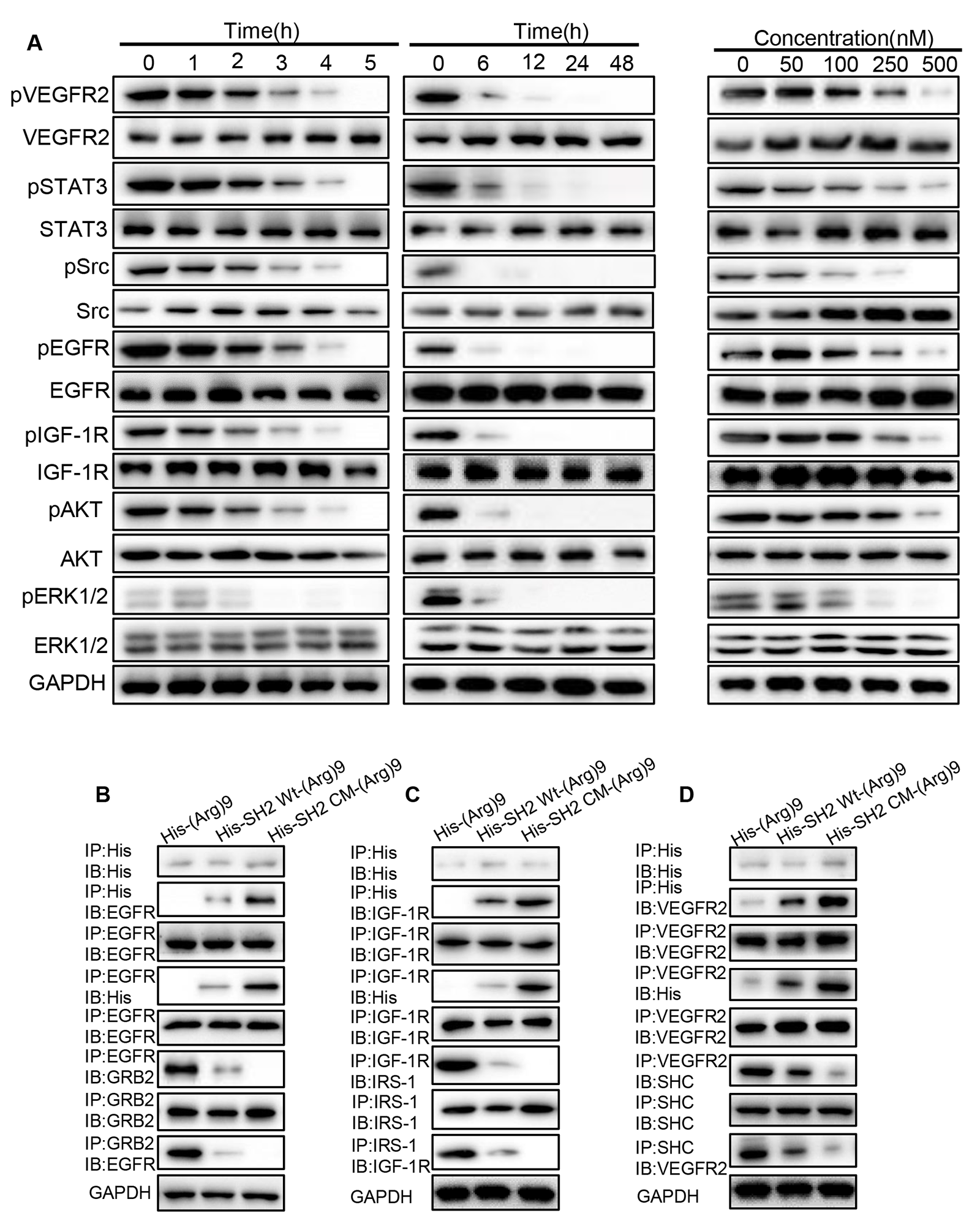

Supplement: Supplementary file 5 — Figure S5. XQ‐2d‐His‐SH2 CM‐(Arg)9 influenced multiple signaling cascades of MiaPaca‐2 cells. (A) Inhibition of EGFR, VEGFR2, IGF‐1R, Src, AKT and ERK1/2 phosphorylation by XQ‐2d‐His‐SH2 CM‐(Arg)9 was examined in MiaPaca‐2 cells. (B‐D) The panels showed reciprocal immunoprecipitation of EGFR and GRB2 (B), IGF‐1R and IRS1(C), VEGFR2 and SHC (D) in MiaPaca‐2 cells treated as indicated above. IP, immunoprecipitation; IB, immunobloting. Data shown are representative of three independent experiments. [file CTM2-11-e337-s005.tif]
