# Supplementary material for: Aptamer‐SH2 superbinder‐based targeted therapy for pancreatic ductal adenocarcinoma
Source: Clin Transl Med. 2021 Feb 26;11(3):e337. doi: 10.1002/ctm2.337 (PMC7908048; doi:10.1002/ctm2.337)
Supplement: Supplementary file 6 — Table S1. Amino acid sequences of Src SH2 domain related variants. Src SH2 TrM contains three amino acid substitutions: T65V, C70A and K88L, shown in bold and red; Src SH2 CM domain contains four amino acid substitutions: T65V, C70A, K88L and C123S, shown in bold and red. Sequence of (Arg)9 is marked in green and bold. Sequence of 6 × His is marked in blue and bold. The recombinant protein was constructed based on the His‐tagged Src SH2 domain triple mutant protein with (Arg)9 at C‐terminus, named His‐SH2 TrM‐(Arg)9. Cysteine residue on site 123 of His‐SH2 TrM‐(Arg)9 was mutated to Serine without affecting the structure and binding property, and the modified SH2 superbinder was named SH2 CM. [file CTM2-11-e337-s006.docx]

Table S1. Sequence of constructs.

|  | Sequence(5’-3’) |
| --- | --- |
| His-Src SH2 Wt-(Arg)_9_ | **HHHHHH**PMSDYDIPTTENLYFQGAMDSIQAEEWYFGKITRRESERLLLNAENPRGTFLVRESET**T**KGAY**C**LSVSDFDNAKGLNVKHY**K**IRKLDSGGFYITSRTQFNSLQQLVAYYSKHADGL**C**HRLTTVCPTSKG**RRRRRRRRR** |
| His-Src SH2 TrM-(Arg)9 | **HHHHHH**PMSDYDIPTTENLYFQGAMDSIQAEEWYFGKITRRESERLLLNAENPRGTFLVRESET**V**KGAY**A**LSVSDFDNAKGLNVKHY**L**IRKLDSGGFYITSRTQFNSLQQLVAYYSKHADGL**C**HRLTTVCPTSKG**RRRRRRRRR** |
| His-Src SH2 CM-(Arg)9 | **HHHHHH**PMSDYDIPTTENLYFQGAMDSIQAEEWYFGKITRRESERLLLNAENPRGTFLVRESET**V**KGAY**A**LSVSDFDNAKGLNVKHY**L**IRKLDSGGFYITSRTQFNSLQQLVAYYSKHADGL**S**HRLTTVCPTSKG**RRRRRRRRR** |
| His-(Arg)9 | **HHHHHH**GGGRRRRRRRRR |
|  |  |

Src SH2 domain and the superbinder were underlined, respectively. Src SH2 TrM contains three amino acid substitutions: T65V, C70A and K88L, shown in **bold** and **red**; Src SH2 CM domain contains four amino acid substitutions: T65V, C70A, K88L and C123S shown in **bold** and **red.** Sequence of (Arg)9 were in green and **bold**. Sequence of 6×His were in **blue** and **bold**. His-(Arg)9 was synthesized by Shanghai Sangon Biotech.
