# Supplementary material for: Aptamer‐SH2 superbinder‐based targeted therapy for pancreatic ductal adenocarcinoma
Source: Clin Transl Med. 2021 Feb 26;11(3):e337. doi: 10.1002/ctm2.337 (PMC7908048; doi:10.1002/ctm2.337)
Supplement: Supplementary file 7 — Table S2. Primer sequences for recombinant plasmids. (Arg)9 was subcloned into pETM11‐SH2 Wt and pETM11‐SH2 TrM by using the One Step Cloning Kit. pETM11‐SH2 CM‐(Arg)9 was constructed by Fast Site‐Directed Mutagenesis Kit. [file CTM2-11-e337-s007.docx]

**Table S2**

**Sequences of Primers**

| **Plasmid** | **Forward Primers****(5’-3’)** | **Reverse Primers(5’-3’)** |
| --- | --- | --- |
| pETM11-  Src SH2 Wt-(Arg)_9_ | TTATTTTCAGGGCGCCATGGACTCCATCCAGGCTGAGG | TGGTGGTGGTGGTGCTCGAGTTAACGGCGGCGACGACGGCGGCGACGACGGCCCTTGGACGTGGGGCACAC |
| pETM11-  Src SH2 TrM-(Arg)_9_ | TTATTTTCAGGGCGCCATGGACTCCATCCAGGCTGAGG | TGGTGGTGGTGGTGCTCGAGTTAACGGCGGCGACGACGGCGGCGACGACGGCCCTTGGACGTGGGGCACAC |
| pETM11-  Src SH2 CM-(Arg)9 | CACGCCGATGGCCTGTCCCACCGCCTCAC | GACAGGCCATCGGCGTGTTTGGAGTAGTAG |
