# Supplementary material for: Aptamer‐SH2 superbinder‐based targeted therapy for pancreatic ductal adenocarcinoma
Source: Clin Transl Med. 2021 Feb 26;11(3):e337. doi: 10.1002/ctm2.337 (PMC7908048; doi:10.1002/ctm2.337)
Supplement: Supplementary file 8 — Table S3. Primer sequences for qPCR. Relative mRNA levels of CD71, VEGF‐A, TGFβ and TNFα were evaluated by qPCR. The Ct value for each sample was normalized based on the control of GAPDH gene. [file CTM2-11-e337-s008.docx]

**Table S3**

**Sequences of Primers**

| **gene** | **Forward Primers****(5’-3’)** | **Reverse Primers(5’-3’)** |
| --- | --- | --- |
| CD71 | AAAATCCGGTGTAGGCACAG | TTAAATGCAGGGACGAAAGG |
| TGFβ1 | CCAGATCCTGTCCAAACTAAGG | CTCTTTAGCATAGTAGTCCGCT |
| TNFα | ATGTCTCAGCCTCTTCTCATTC | GCTTGTCACTCGAATTTTGAGA |
| VEGF-A | TAGAGTACATCTTCAAGCCGTC | CTTTCTTTGGTCTGCATTCACA |
| GAPDH(human) | GGAGTCCACTGGCGTCTTCA | GTCATGAGTCCTTCCACGATACC |
| GAPDH(mouse) | CAGCAACTCCCACTCTTCCAC | TGGTCCAGGGTTTCTTACTC |
